# A Feedback Loop Between Fatty Acid Metabolism and Epigenetics in Clear Cell Renal Carcinoma

**DOI:** 10.1002/advs.202504532

**Published:** 2025-05-20

**Authors:** Zhou Ye, Qi‐Xin Hu, Ming‐Liang Wei, Ji‐Dong Chen, Jia Shi, Ning‐Rong Yang, Lu Jiang, Jian Chen, Zhi‐Yuan Chen, Wei‐Min Yu, Yu Xiao, Kai‐Yu Qian, Zilin Xu, Zhong Wang, Wen‐Lu Qi, Xin‐Yi Xiao, Yu‐Yu Duan, Yong Xiao, Lian‐Yun Li, Lin‐Gao Ju, Ming‐Kai Chen, Min Wu

**Affiliations:** ^1^ State Key Laboratory of Metabolism and Regulation in Complex Organisms, Frontier Science Center for Immunology and Metabolism, Hubei Key Laboratory of Cell Homeostasis, Hubei Key Laboratory of Developmentally Originated Disease, College of Life Sciences, Taikang Center for Life and Medical Sciences, Renmin Hospital of Wuhan University Wuhan University Wuhan Hubei 430072 China; ^2^ Department of Gastroenterology, Renmin Hospital of Wuhan University Wuhan University Wuhan Hubei 430072 China; ^3^ Department of Biological Repositories Zhongnan Hospital of Wuhan University Wuhan 430071 China; ^4^ Department of Urology Renmin Hospital of Wuhan University Wuhan 430060 China; ^5^ Human Genetic Resources Preservation Center of Hubei Province Wuhan 430071 China; ^6^ Hubei University of Chinese Medicine Wuhan Hubei 430065 China

**Keywords:** ccRCC, enhancer, FABP5, H3K27ac, lipid droplet

## Abstract

Lipid storage and epigenetic dysregulation are key features for clear cell renal carcinoma (ccRCC). However, the interplay between fatty acid metabolism and epigenetics in ccRCC remains to be further demonstrated. Here, the landscape of active enhancers is profiled in paired ccRCC samples and identifies 10171 gain variant enhancer loci (VELs) in the tumor tissues. Experimental validation reveals the enhancers targeting *FABP5*, *FABP6*, *LPCAT1*, *MET*, *SEMA5B*, *SH3GL1*, *SNX33*, and *RHBDF2* are oncogenic. Further studies in organoids and animal models prove *FABP5* as an oncogene. HIF‐2α and ZNF692 transcription factors regulate *FABP5* expression through directly binding to its promoter and enhancer. FABP5 is essential for the lipid droplet formation driven by HIFs and critical for H3K27ac and enhancer activity in ccRCC cells. Thus, the study has identified potential targets for drug design and diagnosis and discovered the function of a feedback loop between epigenetics and lipid metabolism regulated by FABP5 in ccRCC.

## Graphical abstract

The current study has profiled the landscape of active enhancers in ccRCC and identified FABP5 as an oncogene. FABP5 is essential for the lipid droplet formation driven by HIFs and critical for H3K27ac and enhancer activity. The study has discovered the function of a feedback loop between epigenetics and lipid metabolism regulated by FABP5 in ccRCC.

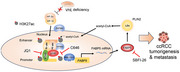

## 1 Introduction

Epigenetic dysregulation is critical for chromatin stability and transcription regulation in cancer cells.^[^ ^1^ ^]^ Transcription factors (TFs) co‐operate with epigenetic modifications to modulate selective transcription.^[^ ^2^, ^3^, ^4^ ^]^ Enhancers reprogramming is tightly associated with transcription regulation in cancer cells.^[^ ^4^, ^5^, ^6^ ^]^ Gain of enhancer activity has been hypothesized to be one of the important features in cancer cells, which is supported by recent studies, and cancer‐specific enhancers are potential novel targets for diagnosis and drug design.^[^ ^2^, ^7^ ^]^ Enhancers are marked by different patterns of histone modifications, which control their activity. H3K4me1 marks primed enhancers; H3K27ac for active enhancers, and H3K27me3 for poised enhancers.^[^ ^3^, ^5^, ^8^ ^]^ H3K27ac ChIP‐Seq has been widely used for the identification of active enhancers in cells and tissues.^[^ ^2^, ^9^ ^]^ However, the key enhancers in many cancers are not well defined.

Clear cell renal cell carcinoma (ccRCC) is the most common type of kidney cancer in adults, and represents ≈80% of all cases.^[^ ^10^, ^11^ ^]^ Formation of lipid droplets (LDs) is one of the key features for ccRCC, but its regulatory mechanisms and the biological impacts remain to be elucidated.^[^ ^10^, ^12^, ^13^ ^]^ LDs are membrane‐less organelles for lipid storage and energy homeostasis, behaving as hubs of cellular metabolism.^[^ ^12^, ^14^ ^]^ Fatty acid (FA) uptake, synthesis, and FA oxidation suppression contribute to lipid storage and LDs formation, which is essential for ccRCC to adapt the environment. In ccRCC, von Hippel–Lindau (VHL) is the most frequently mutated gene, which leads to stabilization of hypoxia‐inducible factor 1α (HIF1α) and activation of HIF transcription factors.^[^ ^15^, ^16^ ^]^ In normal cells, the HIF pathway is activated upon hypoxia conditions, which then causes reprogramming of epigenetics and metabolism.^[^ ^15^ ^]^ In ccRCC cells, HIF pathway mediates the production of multiple genes involved in metabolism, such as solute carrier family 2 member 1 (*SLC2A1*), hexokinase 2 (*HK2*), lactate dehydrogenase A (*LDHA*), glutaminase 1 (*GLS1*), pyruvate dehydrogenase kinase 1 (*PDK1*) and carnitine palmitoyltransferase 1A (*CPT1A*), which increase glycolysis and suppress the entry into the tricarboxylic acid (TCA) cycle.^[^ ^10^, ^17^ ^]^ Thus, it is critical to understand the mechanisms and functions of LD formation in ccRCC.

Epigenetic dysregulation is critical for ccRCC.^[^ ^18^ ^]^ SET domain containing 2 (SETD2), one enzyme for H3K36me3, is frequently mutated, and its protein product is degraded by speckle‐type BTB/POZ protein (SPOP), one oncogenic E3 ligase, in ccRCC.^[^ ^19^, ^20^ ^]^ HIF pathway is also one of the key processes for epigenetic reprogramming in ccRCC. The activation of HIFs drives the expression of several histone H3K9 demethylases, such as lysine demethylase 4A (*KDM4A*), lysine demethylase 4B (*KDM4B*) and lysine demethylase 3A (*KDM3A*), which remove the repressive methylation marks of H3K9 and promote gene transcription.^[^ ^21^ ^]^ Meanwhile, HIFs interact with E1A binding protein p300 (EP300) to loosen chromatin structure and activate transcription.^[^ ^22^ ^]^ It has been reported that epigenetic reprogramming is associated with FA metabolism;^[^ ^23^ ^]^ however, the interplay between them in the ccRCC is not clear.

To understand the epigenetic regulation in ccRCC, we have collected patient paired tissues and performed H3K27ac ChIP analysis to identify oncogenic enhancers. In combination with transcriptomic analysis, we have identified fatty acid binding protein 5 (FABP5) is highly expressed in the tumors, and plays a key role in ccRCC by bridging epigenetic and fatty acid metabolism.

## 2 Results

### 2.1 Transcriptomic and Epigenomic Profiling in ccRCC

**Figure 1:**
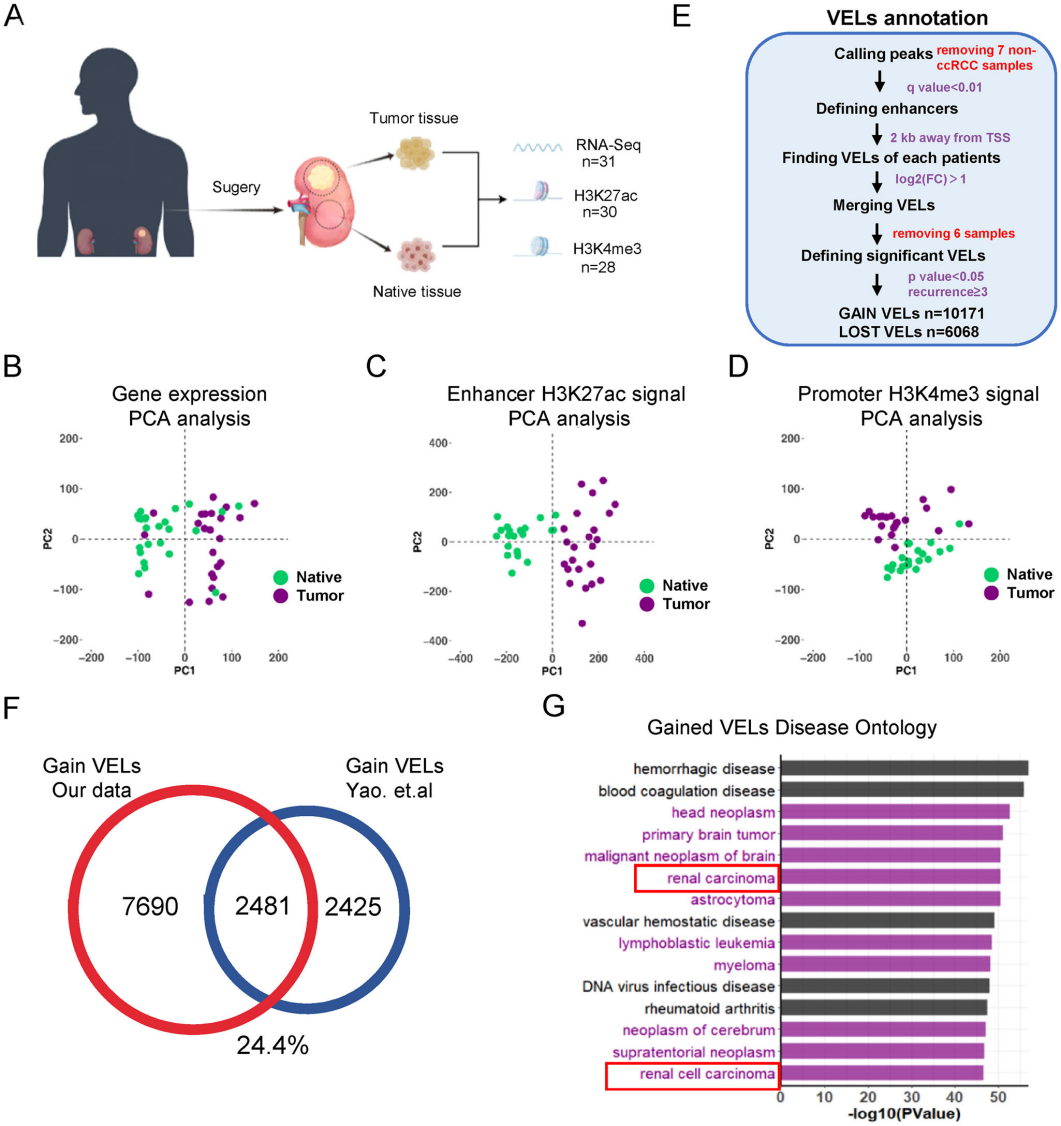
Profiling of tumor‐specific enhancers enriched in ccRCC paired tissues. A) Experimental diagram for studying the enhancer landscapes of tumor and native tissues from ccRCC patient tissues. B–D) PCA analyses to classify tumor and native tissues using gene expression (B), significant enhancers (C), and promoter (D) information identified with our data. E) The comparison between the VELs identified in our studies and one early study.^[^ ^24^ ^]^F) Overlap of enhancer loci between our patient data and 10 primary tumor/normal pairs (GSE86095). G) The disease ontology of the proximal genes of the gain VELs. The purple bars represent cancer‐related diseases, and the black bars represent other diseases.

To investigate the dynamic changes of gene expression and enhancer reprogramming in ccRCC, we collected a cohort of 35 renal carcinoma patients, among which 28 were ccRCC. We then performed RNA‐Seq and ChIP‐Seq analyses in the paired adjacent and tumor specimens, and successfully got transcriptomic profiles of 31 patients, H3K27ac profiles of 30 patients, and H3K4me3 profiles of 28 patients (**Figure** **1**A; Figure S1, Supporting Information). Spearman correlation analysis with transcriptomic, H3K27ac, and H3K4me3 data shows that most of the adjacent tissues grouped together, while those from tumor tissues show higher discrepancy among individuals (Sup. Figure S2A–C, Supporting Information). PCA analyses with the above three types of data divided the adjacent and tumor tissues into different groups, respectively, suggesting the information might be useful in cancer classification. Analysis for different expressed genes (DEGs) identified 4501 upregulated and 4058 downregulated genes in tumors (Figure S2D and Table S1, Supporting Information). H3K27ac distribution across chromatin was then analyzed, and we did not observe a significant difference between the adjacent and tumor tissues (Figure S2E,F, Supporting Information).

### 2.2 Identification of Variant Enhancers in Tumor Tissues

**Figure 2:**
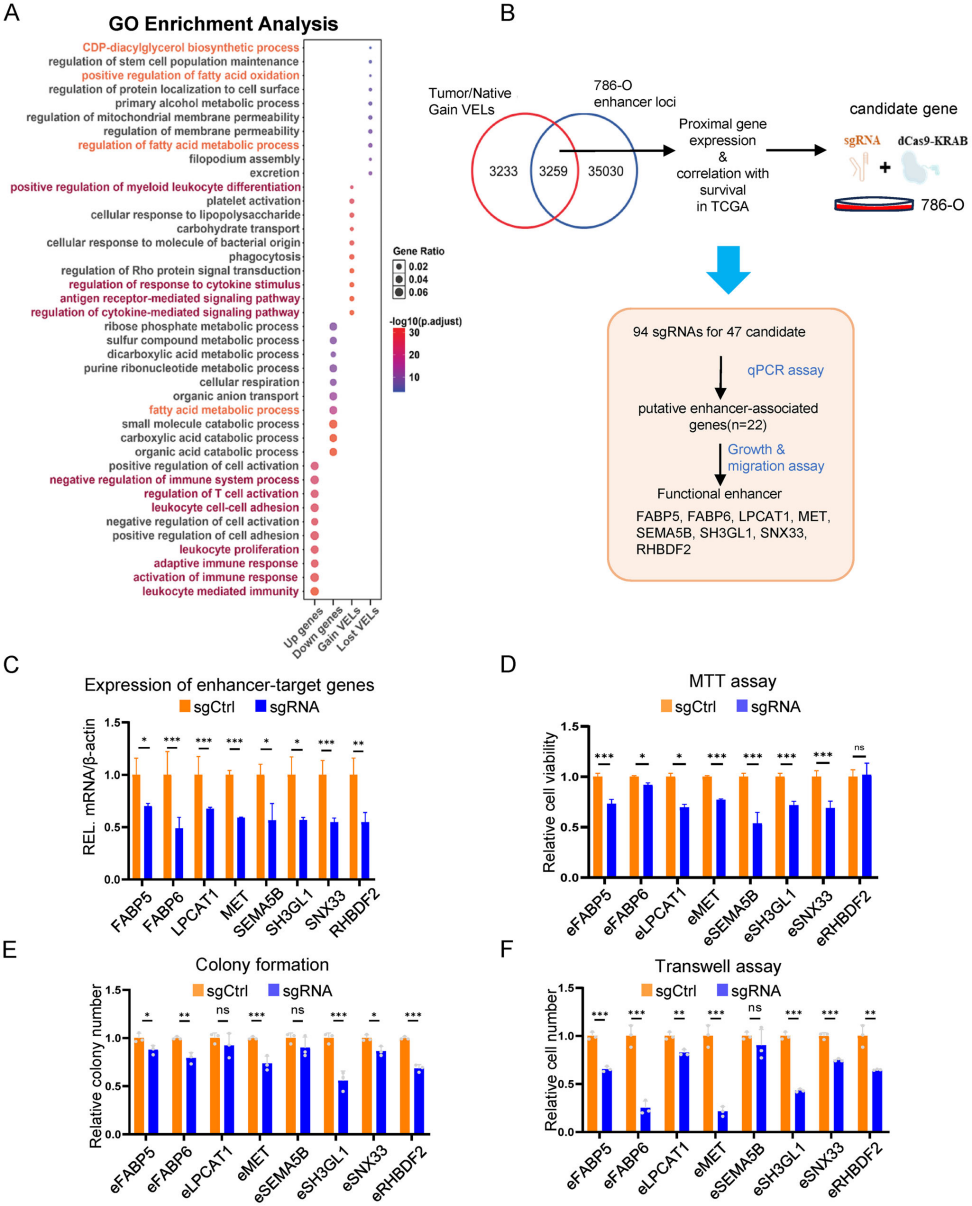
Functional annotation and validation of tumor‐specific enhancers. A) A dot map showing the enriched biological processes of DEGs (log2(FC) > 1 and false discovery rate [FDR] < 0.05) and genes associated with gain and lost of VELs. The dot color and dot size represent–log10(p‐adjust) and gene ratio, respectively. The purple and orange colors highlight the immune and lipid metabolic processes. B) The workflow of functional enhancer screening by the dCas9‐KRAB‐Mecp2 system. C) Relative mRNA level of *FABP5*, *FABP6*, *LPCAT1*, *MET*, *SEMA5B*, *SH3GL1*, *SNX33* and *RHBDF2* in control and sgRNA 786‐O cells (n = 3). D–F) MTT (D), colony formation (E), and transwell assays (F) for 786‐O cells stably transfected with dCas9‐KRAB sgRNAs of the mentioned enhancers (n = 3). Bars represent mean values ± SD, * *p* < 0.05, ** *p* < 0.01, *** *p* < 0.001, two‐sided t‐test.

The distal enhancers were identified according to the H3K27ac peaks far away from transcription start sites (TSS), from which we have identified 10 171 gain variant enhancer loci (VELs) and 6068 lost VELs in tumor tissues (Figure 1F,G; Figure S2G–N and Tables S2 and S3, Supporting Information). Compared with the previous study in ccRCC^[^ ^24^ ^]^ 2/3 of the identified gain VELs are new, which provides an important resource for future studies (Figure 1F). The disease ontology analysis shows that the gain VELs in tumors are enriched in cancer‐related processes (Figure 1G). The GO analysis shows that the up‐regulated DEGs in tumors are enriched mainly in immune response, and the down‐regulated DEGs in metabolic processes (**Figure** **2**A). We took the proximal genes of enhancers as their potential target genes, and GO analysis shows that the functions of the gain and lost VELs are nicely correlated with DEGs (Figure 2A; Figure S2K,L, Supporting Information). Among them, we found that the higher expression of many well‐known oncogenes in ccRCC is associated with up‐regulated enhancer activity, such as *VEGFA* (Figure S2M, Supporting Information).

### 2.3 Prediction of Functional Transcription Factors in ccRCC

To predict the functional transcription factors (TFs) in ccRCC, the DNA sequences of VELs were analyzed by HOMER motif analysis. The top hits of gain and lost VELs were listed (Figure S3A,B, Supporting Information). HIF‐2α was enriched in the motif of gain VELs, which was consistent with the previous study that HIF‐2αcould bind to enhancers to regulate gene transcription in ccRCC. The core regulatory circuitry (CRC) prediction model was also used for the prediction of core TFs in ccRCC tissues (Figure S3C,D, Supporting Information). To verify the key TFs in ccRCC, we surveyed their relationships with patient survival rates according to the TCGA database, and found that the higher expression of iroquois homeobox 3 (*IRX3*), heat shock transcription factor 4 (*HSF4*), RUNX family transcription factor 1 (*RUNX1*), and CCAAT enhancer binding protein beta (*CEBPB*) is associated with low survival rates, suggesting they potentially play oncogenic roles in ccRCC (Figure S3E–H, Supporting Information).

### 2.4 Functional Validation of the Tumor‐Specific VELs

To validate the identified gain VELs, we overlapped the gain VELs with the active enhancers in 786‐O cells, combined the information of their proximal gene expression, and selected a total 47 VELs as candidates. Using the dCas9‐KRAB genome editing tool, we designed two sgRNAs for each enhancer, established stable repressive cell lines, and studied their functions in ccRCC (Figure 2B–F; Figure S4A–J, Supporting Information). The recurrences of some validated VELs are shown (Figure S2N, Supporting Information). The application of sgRNA significantly repressed histone acetylation on the enhancers and the expression of their target genes (Figure 2C; Figure S4I, Supporting Information). An enhancer inhibitor, JQ1, successfully inhibited the expression of most of the target genes, supporting that their expression is regulated by enhancers (Figure S4J, Supporting Information). The functional survey of the identified enhancers indicates that the identified enhancers for *FABP5*, fatty acid binding protein 6 (*FABP6*), lysophosphatidylcholine acyltransferase 1 (*LPCAT1*), MET proto‐oncogene (*MET*), semaphorin 5B (*SEMA5B*), SH3 domain containing GRB2 like 1 (*SH3GL1*) and sorting nexin 33 (*SNX33*) regulate cell viability, enhancers for *FABP5*, *FABP6*, *MET*, *SH3GL1*, *SNX33* and rhomboid 5 homolog 2 (*RHBDF2*) regulate ccRCC cell colony formation, and enhancers for *FABP5*, *FABP6*, *LPCAT1*, *MET*, *SH3GL1*, *SNX33* and *RHBDF2* regulate cell migration (Figure 2D–F; Figure S5A–G, Supporting Information). *FABP5* enhancer is located between *FABP5* and phosphoprotein membrane anchor with glycosphingolipid microdomains 1 (*PAG1*), and is relatively far away from *FABP5* (≈85 kb). Interestingly, enhancer repression only affected *FABP5* but not *PAG1*, indicating the enhancer function is tightly regulated (Figure S4A, Supporting Information).

### 2.5 The Oncogenic Roles of FABP5 in ccRCC

Among the above target genes, *FABP5* and *FABP6* are in the same gene family, which are involved in the binding and transportation of fatty acids.^[^ ^25^ ^]^ Since reprogramming of fatty acid metabolism is one of the distinct features for ccRCC, and *FABP6* is expressed very low in ccRCC according to the transcriptomic data, we selected *FABP5* for further investigations.

**Figure 3:**
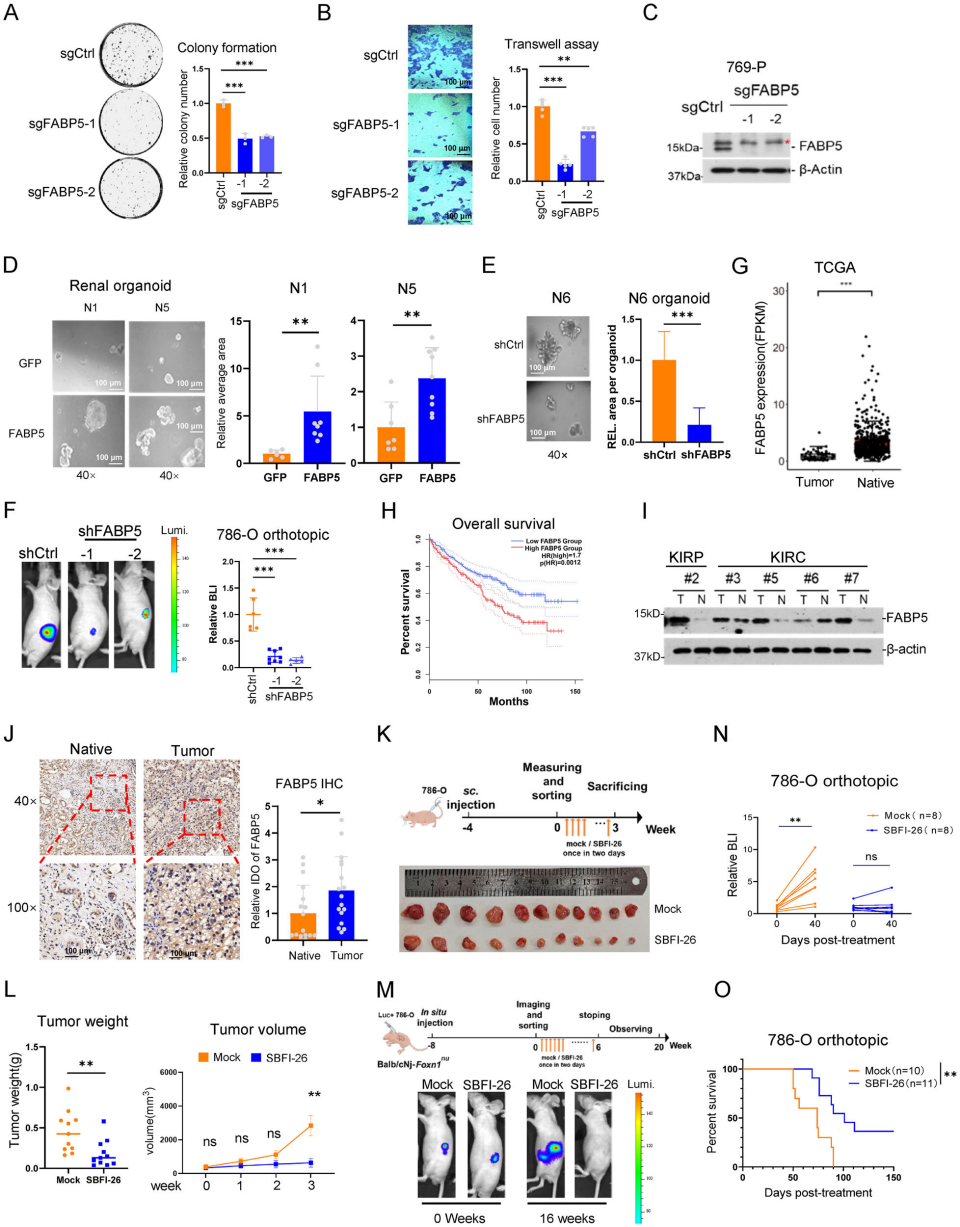
The oncogenic roles of FABP5 in ccRCC. A,B) Colony formation assay (A) and transwell assay(B) of control and *FABP5* KO 769‐P cells. C) Validation of FABP5 knockout by western blotting in 769‐P cells. * represents unspecific bands. D) Representative images and quantification data of kidney organoids transfected with *GFP* or *FABP5*. n = 5. E) Representative images and quantification data of kidney organoids transfected with control or *FABP5* shRNA. n = 5. F) The whole animal bioluminescence (BLI) of control and *FABP5*‐KD 786‐O cells in the immune‐deficient mice 15 weeks after orthotopic injection. The color scale shows photon flux. The right bars show the average animal size; n  =  6 (control), 8 (shFABP5‐1), 5 (shFABP5‐2). G) The mRNA level (RPKM) of *FABP5* (left) in the native and tumor tissues of ccRCC patients from the TCGA database. H) Overall survival rates of *FABP5* high‐ and low‐expressed KIRC patients from TCGA, log‐rank test, n = 516 patients. I) Western blotting to measure FABP5 level in the paired ccRCC tissues. J) Representative image and quantitative data of FABP5 IHC staining. n = 17. K) Experimental diagram (up) and tumor images (bottom, n = 11) of intra‐tumoral SBFI‐26 injection to subcutaneous tumors in immune‐deficient mice. L) Weight and volume of subcutaneous tumor in Figure 3K. M) Experimental diagram (up) and animal bioluminescence (BLI) images (bottom) of intraperitoneal SBFI‐26 injection to orthotopic tumors in immune‐deficient mice. N) Survival of Balb/c nude mice upon in situ injection of Luc+ 786‐O cells with SBFI‐26 or control. O) Quantification of mice upon in situ injection of Luc+ 786‐O cells with SBFI‐26 or control at the indicated time. Bars represent mean values ± SD, * *p* < 0.05, ** *p* < 0.01, *** *p* < 0.001, two‐sided *t*‐test.

We knocked down *FABP5* in 769‐P cells by two different sgRNAs, respectively, which significantly reduced the colony number and cell migration (**Figure** **3**A). Repressed expression by shRNAs in multiple cell lines also showed similar results, while exogenous expression exhibited the opposite (Figure S6A–Q, Supporting Information). We cultured renal organoids from the adjacent tissues from ccRCC patients. *FABP5* expression in the organoids significantly increased the organoid sizes, while *FABP5* knockdown via shRNA repressed organoid growth (Figure 3D,E). To examine the role of *FABP5* in vivo, we orthotopically injected control and *FABP5* knockdown 786‐O cells into the kidneys of the immune‐deficient mice. The result showed that *FABP5* deficiency significantly repressed tumor growth in vivo (Figure 3F). According to the information in the TCGA database, *FABP5* was expressed higher in the ccRCC tumors compared with the native tissues; the patients with higher *FABP5* expression also exhibited a low survival rate (Figure 3G,H). We randomly selected some collected ccRCC specimens and checked FABP5 expression using western blotting, in most of which showed higher FABP5 expression compared with their corresponding adjacent tissues (Figure 3I). Patient #6 showed low *FABP5* expression level in the tumor (Figure 3I); later we found that its VHL was wild type, which suggested that *FABP5* expression is possibly related with HIF pathway. Immunohistochemical staining also showed that FABP5 was highly expressed in ccRCC (Figure 3J). These indicate that *FABP5* is an oncogene in ccRCC.

### 2.6 Inhibition of FABP5 Activity by SBFI‐26 Represses ccRCC

To explore the potential application by modulating FABP5 activity, we used SBFI‐26, a small molecular chemical inhibitor for FABP5.^[^ ^26^ ^]^ SBFI‐26 significantly repressed 786‐O proliferation in a dose‐dependent manner in vitro (Figure S6R, Supporting Information) and repressed tumor growth in vivo both in the subcutaneous and orthotopic xenograft models (Figure 3K–N). Mice treated with SBFI‐26 exhibited significantly prolonged survival (Figure 3O). Thus, FABP5 is a new drug target, and SBFI‐26 could be a potential drug for ccRCC treatment.

### 2.7 Regulation of *FABP5* Expression by VHL/HIF Pathway

**Figure 4:**
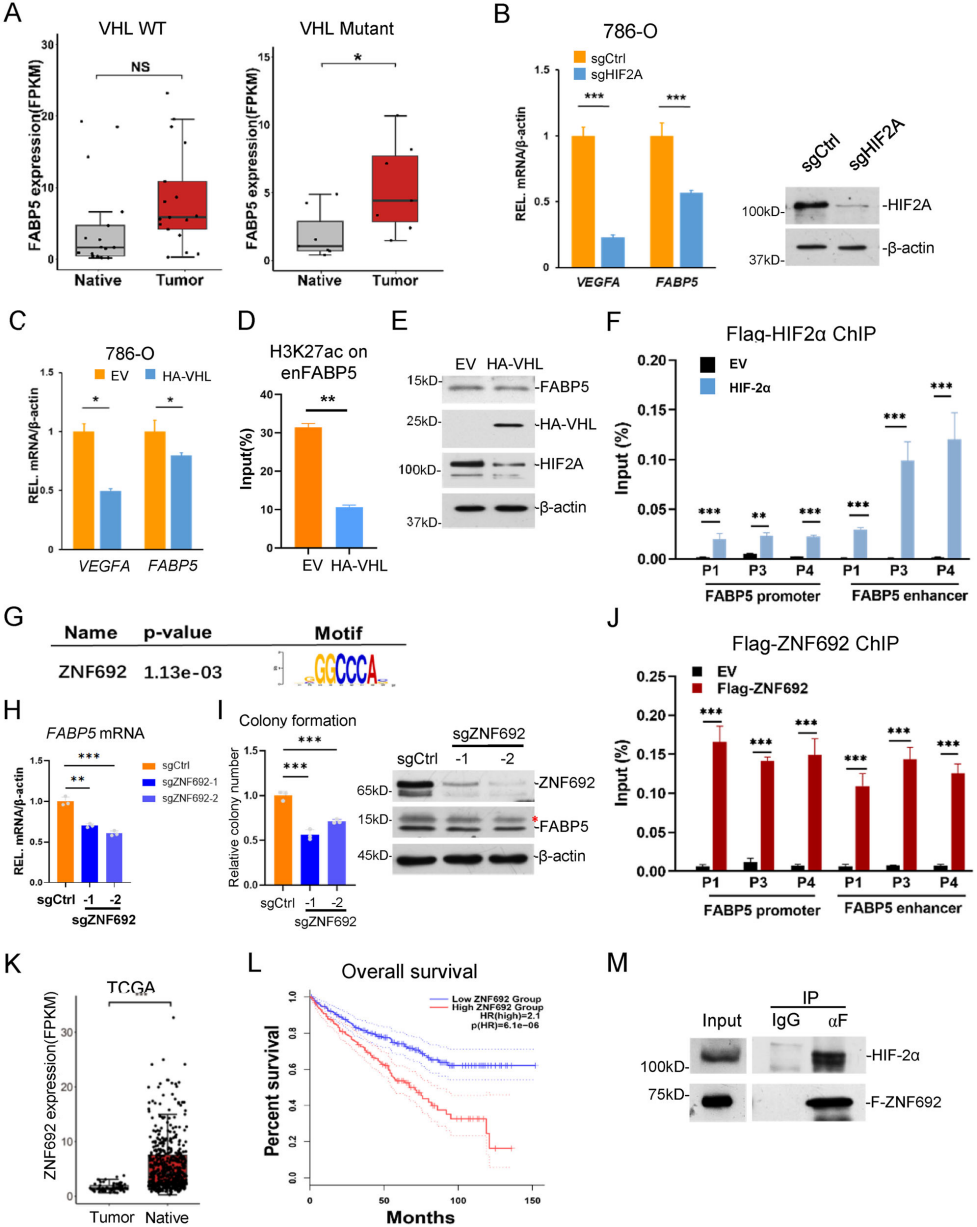
Transcriptional regulation of *FABP5* by VHL/HIF and ZNF692. A) *FABP5* expression in the normal and tumor tissues with wild‐type *VHL* (left) or mutant *VHL* (right) in the ccRCC patient tissues. n = 18 for WT, 6 for mutant. B–D) Relative mRNA level of *VEGFA* and *FABP5* (A), and H3K27ac enrichment on FABP5 enhancer site (B) in control and pVHL 786‐O cells. n = 3. The indicated proteins were measured with western blotting. E) Relative mRNA level of *VEGFA* and *FABP5* in control and *HIF2A*‐KO 786‐O cells. n = 3. F) HIF‐2α enrichment on *FABP5* promoter and enhancer sites in 786‐O detected by ChIP‐qPCR. G) Consensus sequence of the ZNF692 binding motif. H) Relative mRNA level of *FABP5* in control and *ZNF692*‐KO 786‐O cells. I) Colony formation assay of control and *ZNF692*‐KO 786‐O cells. J) ZNF692 enrichment on *FABP5* promoter and enhancer sites in 786‐O transfected with Flag‐ZNF692 detected by ChIP‐qPCR. K) mRNA level (RPKM) of *ZNF692* in native and tumor tissues of ccRCC patients from the TCGA database. L) Overall survival rates of patients with KIRC from TCGA stratified based on tumor *ZNF692* expression intensity, log‐rank test, n = 516. M) Co‐immunoprecipitation with anti‐Flag in Flag‐ZNF‐692 stably expressed 293T cells and western blotting with the indicated antibodies.

*VHL* mutation and HIF pathway activation frequently occur in ccRCC, and our previous result showed a potential link between FABP5 and *VHL* mutation (Figure 3I). To investigate the mechanism for FABP5 activation in ccRCC, we first studied the relationship between *FABP5* transcription and HIF activation. Based on our datasets, we found that *FABP5* mRNA expression showed a significant difference between the adjacent and tumor tissues with *VHL* mutation, but not in the *VHL* wild‐type samples (**Figure** **4**A). H3K27ac enrichment on *FABP5* enhancer both increased in *VHL* wild type and mutant groups, but with less fold changes in wild‐type (Figure S7A,B, Supporting Information). *HIF2A* knockdown with CRISPR significantly decreased *FABP5* expression (Figure 4B). Exogenous expression of *VHL* in 786‐O cells significantly down‐regulated *FABP5* expression, as well as the H3K27ac enrichment on *FABP5* enhancer (Figure 4C–E). To investigate whether HIF‐2α directly regulates *FABP5*, we established one cell line stably expressing Flag‐tagged HIF‐2α. ChIP‐qPCR with anti‐Flag showed that HIF‐2α bound on the *FABP5* promoter and enhancer (Figure 4F). These indicate that HIF TFs directly regulate *FABP5* transcription.

### 2.8 ZNF692 is a Critical Transcription Factor for FABP5

Since the *FABP5* enhancer had higher H3K27ac level even in *VHL* wild type tumors (Figure S7A,B, Supporting Information), we investigated whether *FABP5* is regulated by other TFs. we analyzed the sequence of *FABP5* enhancer, which predicted another potential TF, zinc finger protein 692 (ZNF692) (Figure 4G). ZNF692 is a TF containing zinc fingers whose function is not well characterized. We knocked down *ZNF692* in 786‐O cells, which resulted in lower expression of *FABP5* mRNA and repressed cell proliferation, migration, and colony formation (Figure 4H,I; Figure S7C,D, Supporting Information). ChIP‐qPCR in a Flag‐ZNF692 stable cell line showed that Flag‐ZFN692 bound both on *FABP5* promoter and enhancer (Figure 4J). Analysis with TCGA datasets showed that *ZNF692* was expressed higher in tumor tissues, and its high expression was associated with low survival rate (Figure 4K,L). Interestingly, western blotting with patient specimens showed two different bands for ZNF692 specific in tumor tissues, while the adjacent tissues only contained one small form (Figure S7E, Supporting Information), suggesting alternative splicing of the transcripts or post‐translational regulations on proteins might exist for *ZNF692* in ccRCC cells. Co‐immunoprecipitation assay in Flag‐ZNF692 stable cells showed that ZNF692 interacted with HIF‐2α (Figure 4M). These results together indicate that ZNF692 plays an oncogenic role in ccRCC through interaction with HIF‐2α and regulating *FABP5* expression.

### 2.9 FABP5 is Critical for Lipid Accumulation in ccRCC Cells

**Figure 5:**
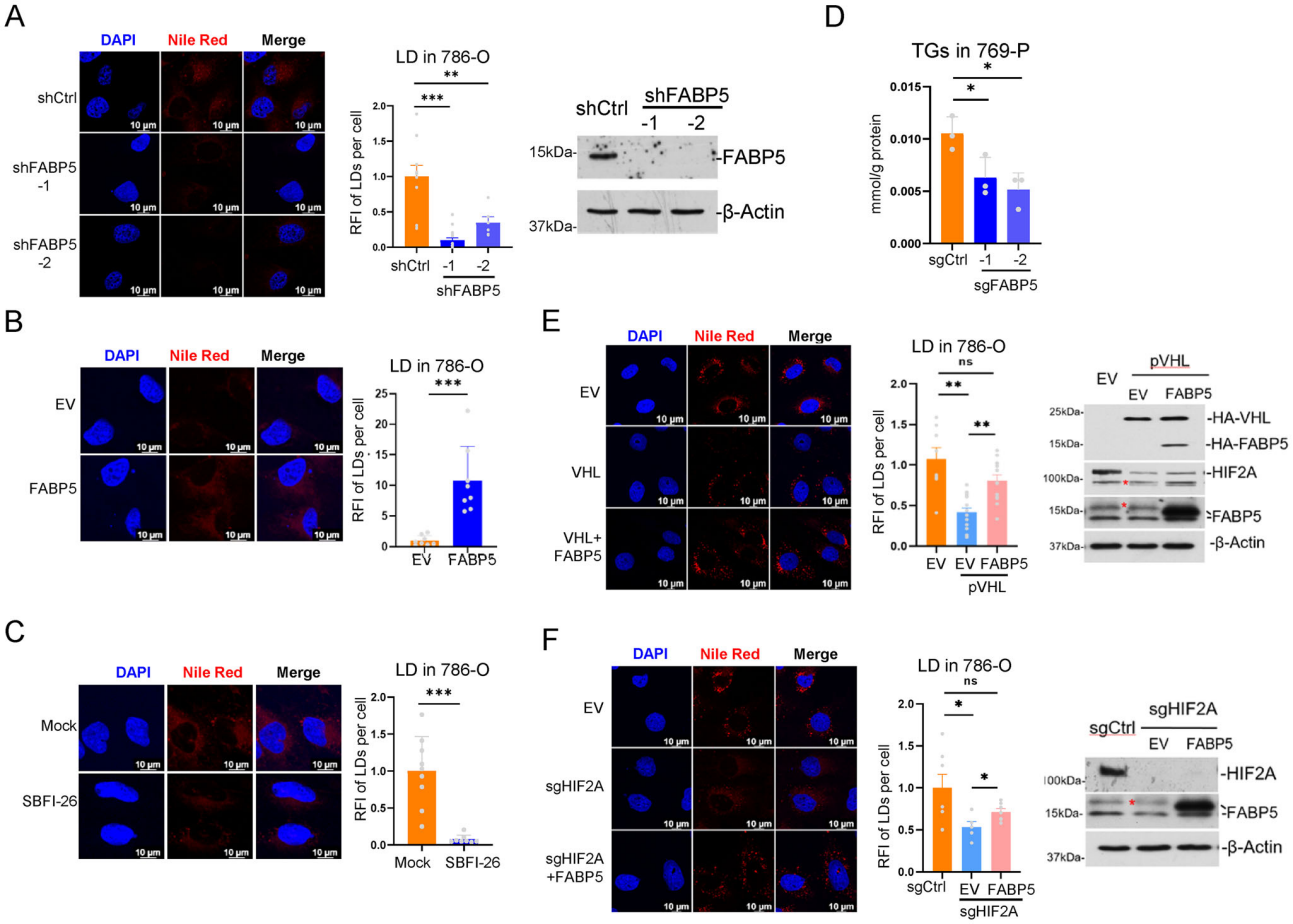
FABP5 is indispensable for lipid accumulation driven by the HIF pathway. A,B) Representative images and statistical results for LD (red) and nuclei (blue) in *FABP5*‐KO 769‐P cells (A), or *FABP5* stably expressed cells (B). C) 786‐O cells were treated with 100 µM SBFI‐26 for 36 h. Representative images for LD (red) and nuclei (blue), and statistical results are shown. n = 9 for DMSO, 8 for SBFI‐26. D) Measurement of triglyceride in control and *FABP5* KO 769‐P cells (n = 3). E) *VHL* was exogenous expressed alone or together with *FABP5* in A498 cells. Representative images and statistical results for LD (red) and nuclei (blue) were shown. n = 7 (EV), 11 (*VHL*), 10 (*VHL*+*FABP5*). Western were performed as indicated. F) *HIF2A* was knocked down w/wo *FABP5* expression in A498 cells. Representative fluorescence images and quantitative data of the indicated A498 cells stained for LD (red) and nuclei (blue). n = 6. Bars represent mean values ± SD, * *p* < 0.05, ** *p* < 0.01, *** *p* < 0.001, two‐sided t‐test.

After determining the mechanisms for *FABP5* transcription, we continued to investigate how FABP5 promotes ccRCC. FABP5 has been shown to regulate fatty acid accumulation in the cytosol.^[^ ^27^ ^]^ We found that *FABP5* knockdown, either by shRNAs or sgRNAs, in 769‐P cells significantly reduced the amount of LDs, while *FABP5* expression increased them (**Figure** **5**A,B; Figure S8A,B, Supporting Information). Inhibition of FABP5 by SBFI‐26 also significantly reduced LD amount in ccRCC cells (Figure 5C; Figure S8C, Supporting Information), and repression of *FABP5* enhancer showed a similar phenotype (Figure S8D, Supporting Information). *FABP5* deficiency also decreased the amount of triglyceride in the cells (Figure 5D). HIF pathway activation is critical for lipid accumulation in ccRCC cells.^[^ ^10^ ^]^ *VHL* expression or *HIF2A* knockdown eliminated the LD signal in cells, while *FABP5* expression is sufficient to rescue the phenotype (Figure 5E,F). These indicate that in ccRCC, FABP5 is the key player for fatty acid metabolism downstream of the HIF pathway.

### 2.10 FABP5 Regulates Histone Acetylation in ccRCC Cells

**Figure 6:**
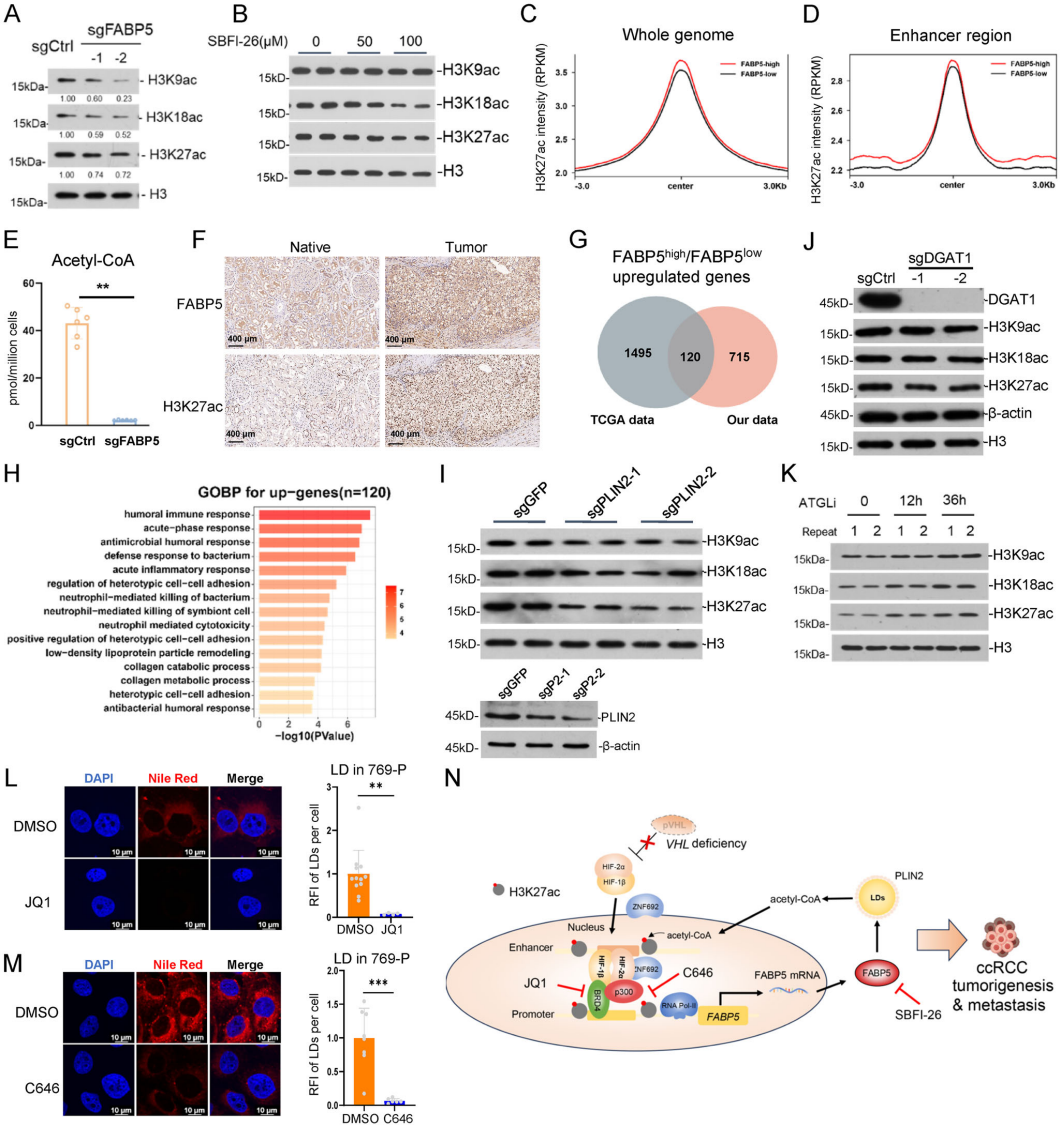
The roles of FABP5 in the crosstalk between lipid metabolism and epigenetic reprogramming. A) Histone acetylation on K9, K18, and K27 of control and *FABP5* KO 769‐P cells detected by WB. B) Histone acetylation of 769‐P cells treated with 50 or 100 µM SBFI‐26 for 36 h. C,D) The average H3K27ac signal (RPKM) on the whole genome (C) and enhancer regions (D) in *FABP5* ^high^ versus *FABP5* ^low^ tumor tissues. E) Measurement of acetyl‐CoA in control or *FABP5*‐KO 769‐P cells by mass spec (n = 6). F) IHC staining of FABP5 and H3K27ac in ccRCC tissues. G) Overlapped upregulated genes in *FABP5* ^high^ versus *FABP5* ^low^ tumor tissues between TCGA and our patients’ data. H) GO analysis of overlapped genes in (F). I,J) *PLIN2* (I) or *DGAT1* (J) was knocked down in 769‐P cells, and western blotting was performed with the indicated antibodies. K) 769‐P cells were treated with ATGL inhibitor for the indicated time, and western blotting was performed with the indicated antibodies. L) Representative fluorescence images and statistical data of indicated 769‐P cells treated with 5 µM JQ1 for 36 h, followed by staining for lipid droplets (red) and nuclei (blue). n = 12 (DMSO) or 4 (JQ1). M) Representative fluorescence images and statistical analysis of 769‐P cells treated with 5 µM C646 for 36 h, followed by staining for lipid droplets (red) and nuclei (blue). n = 8 (DMSO) or 6 (C646). N) A sketch for the interplay between lipid droplets regulated by FABP5 and epigenetics in ccRCC. Bars represent mean values ± SD, * *p* < 0.05, ** *p* < 0.01, *** *p* < 0.001, two‐sided t‐test.

Since lipid metabolism is tightly associated with acetyl‐CoA production and protein acetylation, we investigated whether FABP5 regulates histone acetylation and the related epigenetic events. In the *FABP5* knockdown 769‐P cells, the level of H3K9ac, H3K18ac, and H3K27ac significantly decreased (**Figure** **6**A), and the treatment with SBFI‐26 showed a similar effect (Figure 6B). In the ccRCC tissues with higher *FABP5* expression, their H3K27ac enrichment was higher compared with the *FABP5*‐low group, both on the whole genome and enhancer regions (Figure 6C,D). Mass spectrometry analysis showed that acetyl‐CoA greatly decreased in the *FABP5*‐deficient cells (Figure 6E). Immunohistochemistry (IHC) staining in ccRCC tissues showed that the high H3K27ac level was correlated with high FABP5 expression (Figure 6F). These indicate that FABP5 positively regulates histone acetylation, probably through acetyl‐CoA. We analyzed the DEGs between *FABP5*‐high and *FABP5*‐low tumors, using both the TCGA and our datasets. We overlapped the two sets of DEGs and obtained the potential FABP5‐regulating genes (Figure 6G). GO analysis showed that these genes were enriched in immune, lipid metabolism, and cell adhesion pathways (Figure 6H). Taken together, these suggest that FABP5 possibly regulates immunity, lipid metabolism, and extracellular environment in ccRCC through modulating metabolism and epigenetics.

### 2.11 Association Between Lipid Droplet Accumulation and Histone Acetylation

To further investigate the relationship between LDs and histone acetylation in ccRCC, we knocked down perilipin 2 (*PLIN2*) in the cells, an essential protein for LD formation. *PLIN2* deficiency caused the disappearance of LD signal in the cytosol, as well as down regulation of histone acetylation (Figure 6I; Figure S9A, Supporting Information). Diacylglycerol O‐acyltransferase 1 (DGAT1) is the key enzyme for triacylglycerol synthesis, and critical for LD formation.^[^ ^10^, ^12^ ^]^ *DGAT1* knockdown also decreased LD amount and histone acetylation (Figure 6J; Figure S9B, Supporting Information). Patatin like phospholipase domain containing 2 (PNPLA2/ATGL) is the key factor for fatty acid hydrolysis.^[^ ^10^, ^12^ ^]^ The application of ATGL inhibitor significantly increased LD amount, and increased histone acetylation (Figure 6K; Figure S9C, Supporting Information). Taken together, it seems that LD amount is associated with histone acetylation level, and the detailed mechanisms require further investigation.

To investigate whether histone acetylation regulates LD formation, we treated the cells with JQ1, one inhibitor for BRD4 and enhancers, or with C646, one inhibitor for histone acetylase p300, and found that both drugs repressed LDs in 796‐P and 786‐O cells (Figure 6L,M; Figure S9D,E, Supporting Information). We also knocked down p300 in the cells, which showed a similar phenotype (Figure S9F, Supporting Information). These indicate that histone acetylation and enhancer activity are also important for lipid accumulation in ccRCC, probably through their functions in transcription regulation.

## 3 Discussion

Epigenetic dysregulation is one of the features for multiple cancers. In the current study, we have profiled the dynamic active enhancers in the paired ccRCC tissues and identified 10 171 gain VELs in ccRCC, ≈2/3 are new enhancers compared with the previous study.^[^ ^24^ ^]^ We then experimentally verified the oncogenic roles of the enhancers for *FABP5, FABP6*, *LPCAT1*, *MET*, *SEMA5B*, *SH3GL1*, *SNX33*, and *RHBDF2* in ccRCC cells, and predicted IRX3, HSF4, RUNX1, and CEBPB as potential oncogenic TFs. These provide important information for future studies.

FABP5 is one protein critical for fatty acid binding and metabolism. It has been reported to be involved in the sensing of dietary ω‐6 linoleic acid and the pathological progression of metabolic disorders, breast cancer, and hepatocellular carcinoma.^[^ ^28^ ^]^ Here we have identified *FABP5* as an oncogene in ccRCC, which promotes ccRCC cell proliferation, migration, colony formation, organoid, and tumor growth. *FABP5* is highly expressed in ccRCC tissues, and its high expression is correlated with worse prognosis. Inhibition by a small molecular chemical inhibitor, SBFI‐26, also significantly ccRCC in vitro and in vivo. FABP5 and active enhancers can serve as potential targets for drug design in ccRCC. Our study supports the previous reports and has revealed the new roles of FABP5, which may contribute to the drug design and clinical treatment in ccRCC in the future.

*VHL* is frequently mutated in ccRCC, and activation of the HIF pathway is one of the key signatures for ccRCC. We found that the *FABP5* promoter and enhancer are bound by HIF‐2α, and *FABP5* is one of the target genes for HIFs. HIF activation is critical for fatty acid metabolism and LD formation, one of the key features for ccRCC. In our study, FABP5 expression increased LD formation in ccRCC cells and rescued the LD‐reduction phenotype caused by HIF repression. Our results indicate that FABP5 is one of the key factors for LD regulation downstream of the HIF pathway, which is essential for the reprogramming of fatty acid metabolism in ccRCC. We have also examined the other identified enhancers on LDs (data not shown); enhancers for *SH3GL1*, *SNX33*, and *RHBDF2* have mild impacts, but not comparable with *FABP5*.

Our results have also shown that *FABP5* is transcriptionally regulated by transcription factor ZNF692. ZNF692 binds to the *FABP5* enhancer and promoter and interacts with HIF‐2α. *ZNF692* is expressed higher in ccRCC tissues than the normal tissues, and its high expression is associated with worse prognosis. All these support that ZNF692 is an important oncogenic TF in ccRCC. Recently, *ZNF692* has been reported as an oncogene in several cancer types.^[^ ^29^ ^]^ It is important to further demonstrate its functions in ccRCC and the detailed mechanism in the future.

Metabolic switch and epigenetic reprogramming are considered as key signatures in cancer cells, and are believed to be tightly associated.^[^ ^10^ ^]^ However, the detailed mechanistic studies about their interplay in specific cancer types are still limited. FABP5 behaves as a critical regulator for both lipid accumulation and histone acetylation in ccRCC cells. Moreover, our results show that the increased LD amount is associated with the up‐regulation of histone acetylation level. On the other hand, repression of p300 and enhancer functions led to a reduction of LD. It seems that a positive feedback loop between enhancer regulation and lipid metabolism is formed to facilitate ccRCC tumor growth and development (Figure 6N). It will be important in the future to disrupt the feedback loop for drug design targeting ccRCC. Our study reveals that FABP5 knockout reduces acetyl‐CoA levels. Although this could be linked to FAO due to its role in acetyl‐CoA production, in ccRCC, FAO is generally considered to be repressed through down‐regulation of *CPT1A* expression.^[^ ^10^ ^]^ We have knocked out *ATGL*, one of the key enzymes for lipolysis, and have not observed its impact to histone acetylation. It suggests that a new, unknown mechanism is involved, which requires further investigation.

## 4 Experimental Section

### Reagents and Cell Lines

HEK‐293T cell lines were cultured in Dulbecco's Modified Eagle Medium (DMEM, Gibco) supplemented with 10% fetal bovine serum (FBS, ExCell Bio), 1% penicillin and streptomycin (Hyclone) at 37 °C with 5% CO_2_. 769‐P, 786‐O, and A498 cell lines were cultured in RPMI 1640 (Gibco) supplemented with 10% FBS, 1% penicillin, and streptomycin at 37 °C with 5% CO_2_. All cell lines were purchased from the Cell Bank of the Chinese Academy of Science. Mycoplasma detection was performed every month, and no mycoplasma was detected.

Antibodies were shown in Table S4 (Supporting Information). PCR primers, sgRNA, and shRNA were custom‐synthesized by Tsingke Biotechnology (Tables S5 and S6, Supporting Information).

### ChIP Assay

ChIP assay was performed as previously described.^[^ ^20^, ^30^ ^]^ Briefly, cells were scraped down, collected with centrifugation. Around 60 mg tissues were cut into 1 mm^3^ pieces in PBS with protease inhibitor. The samples were cross‐linked for 10 min at room temperature with 1% formaldehyde and then quenched with 0.125 M of glycine for 5 min. The pellet was washed three times with PBS and then resuspended with digestion buffer (50 mM Tris‐HCl pH 8.0, 1 mM CaCl_2_, 0.2% Triton X‐100). Chromatin was digested with MNase (NEB, M0247S) at 37 °C for 20 min and quenched with 1 mM EDTA. The resulting mixture was sonicated, and immunoprecipitation was performed with 400 µL supernatant, 2 µg antibody, and 200 µL dilution buffer (20 mM Tris‐HCl pH 8.0, 150 mM NaCl, 2 mM EDTA, 1% Triton X‐100, 0.3% SDS) overnight at 4 °C, 40 µL supernatant as input. The beads were washed once with Wash buffer I (20 mM Tris‐HCl pH 8.0, 150 mM NaCl, 2 mM EDTA, 1% Triton X‐100, 0.1% SDS), Wash buffer II (20 mM Tris‐HCl pH 8.0, 500 mM NaCl, 2 mM EDTA, 1% Triton X‐100, 0.1% SDS), Wash buffer III (10 mM Tris‐HCl pH 8.0, 250 mM LiCl, 1 mM EDTA, 1% Na‐deoxycholate, 1% NP‐40) and twice with TE (10 mM Tris‐HCl pH8.0, 1 mM EDTA). The beads were eluted twice with 150 µL elution buffer (1% SDS, 0.1 M NaHCO3, 20 mg mL^−1^ Proteinase K) at room temperature. The elution was incubated at 65 °C for overnight and then purified with a DNA purification kit (TIANGEN DP214‐03). Primers for ChIP‐qPCR were listed in Table S5 (Supporting Information).

### Library Preparation for ChIP‐Sequencing

ChIP‐seq libraries were constructed with ChIP and input DNA using the VATHS Universal DNA Library Prep Kit for Illumina (Vazyme ND606). Briefly, 50 µL of DNA (8–10 ng) was end‐repaired for dA tailing, followed by adaptor ligation. Each adaptor was marked with a barcode of 8 bp DNA. Adaptor‐ligated DNA was purified by AMPure XP beads (1:1) and then amplified by PCR of 9 cycles with the primer matching with adaptor universal part. Amplified DNA was purified again using AMPure XP beads (1:1) in 35 µL EB elution buffer. For multiplexing, libraries with different barcodes were mixed with equal molar quantities (30–50 million reads per library). Libraries were sequenced by the Illumina Nova‐seq platform with pair‐end reads of 150 bp.

### Reverse Transcription and Quantitative PCR

Cells were scraped down and collected with centrifugation. For tissue RNA extraction, 20 mg of tissue was homogenized and collected with centrifugation. Total RNA was extracted with RNA extraction kit (Aidlab or CWBIO) according to the manufacturer's manual. ≈1 µg of total RNA was used for reverse transcription with a RT Master Mix (ABclonal, RK20429). The resulting cDNA was assayed with quantitative PCR, β‐actin or 18S RNA for normalization. The sequences of primers are in Table S5 (Supporting Information). Assays were repeated at least three times. Data were shown as average values ± SD or SEM of at least three representative experiments. P‐value was calculated using student's *t*‐test.

### RNA‐Sequencing

RNA‐seq libraries were constructed by NEBNext Poly(A) mRNA Magnetic Isolation Module (NEB E7490) and NEBNext Ultra II Non‐Directional RNA Second Strand Synthesis Module (NEB E6111). mRNA was purified with poly‐T magnetic beads, and first and second‐strand cDNA were synthesized. The resulting cDNA was purified by AMPure XP beads (1:1) and eluted in 50 µL nucleotide‐free water. The subsequent procedures were the same as described in ChIP‐seq library construction, except that the sequencing depth was 20 million reads per library. RNA‐seq libraries were sequenced by the Illumina Nova‐seq platform with pair‐end reads of 150 bp.

### ChIP‐Seq Data Analysis

ChIP‐seq raw fastq data were quality controlled using FastQC (version 0.11.9, https://www.bioinformatics.babraham.ac.uk/projects/fastqc/). Clean data were obtained by removing adapters with fastp (version 0.21.0, https://github.com/OpenGene/fastp, parameters were “‐f 5 ‐t 20 ‐F 5 ‐T 20 ‐l 30”). Cleaned reads were aligned in paired‐end mode to homo sapiens UCSC reference genome hg19 with BWA mem (version 0.7.12, http://bio‐bwa.sourceforge.net).^[^ ^31^ ^]^ Duplicate reads were removed by picard Markduplicates (version 2.18.29, https://broadinstitute.github.io/picard/). All mapped reads were used in the analysis. Peaks were called by MACS2 (version 2.1.1, https://github.com/taoliu/MACS, parameters “–nomodel ‐p 1E‐7 ‐B –broad –extsize 147”).^[^ ^32^ ^]^ For the comparison in enhancers, a signal of modification in each enhancer was normalized by reads per kilobase per million mapped reads (RPKM).

### RNA‐Seq Data Analysis

RNA‐seq clean data were obtained same as ChIP‐seq data (parameters were “‐f 5 ‐t ‐20 ‐F 5 ‐T ‐20 ‐l 30”). Quality control was done with FastQC (version 0.11.9, https://www.bioinformatics.babraham.ac.uk/projects/fastqc/) and Multiqc (version 1.10.1, https://multiqc.info). Clean reads were aligned to homo sapiens hg19 genome using HISAT2 (version 2.2.1, https://daehwankimlab.github.io/hisat2/). The gene expression level was normalized as fragments per kilobase of bin per million mapped reads (FPKM). Uniquely aligned reads were counted at gene regions using the package featureCounts (version 2.0.6) based on Gencode v19 annotations. Differential gene expression analysis was performed using the R package DESEQ2 (version 1.40.1, https://bioconductor.org/pack‐ages/release/bioc/html/DESeq2.html). Genes whose log2FC ≤ 1 and padj ≤ 1E−2 were identified as differential expressed genes (DEGs).

### Identification of VELs (Variant Enhancer Loci)

To identify the significant variant enhancer loci between native and tumor tissues, all VELs were first identified in paired native and tumor tissues, defined as enhancers whose H3K27ac fold change (FC) ≥ 2 between native and tumor tissues. We merged all VELs into one single coordinate file and calculated the recurrence and significance (Benjamini & Hochberg corrected p‐value) for all VELs. We used a recurrence of 3 as a significance threshold for gain and lost VELs, respectively, because gain and lost VELs achieved the significant percentage cut‐off (0.85) when recurrence was larger than three.

### Transcriptional Factor Enrichment Analysis

We used the findMotifsGenome.pl module in HOMER (version 4.11, http://homer.ucsd.edu/homer/, parameters were ‐size 200 ‐mask) to identify **transcriptional factor** motifs.

### Human Disease Ontology and Gene Ontology Analysis

The coordinate file of gain and lost VELs was submitted to GREAT website (version 3.0.0), and the results of human disease ontology and GO analysis (biological process) were obtained for plotting.

### ChIP‐Seq and RNA‐Seq Data Visualization

UCSC genomic tracks for histone marks, RNA expression, and chromatin state beyond the RefSeq gene model were drawn by karyoploteR^[^ ^33^ ^]^ using the alignment file of chromatin markers and state annotation bed files produced by ChromHMM. Histone marker's signal density panel across TSS and gene body was plotted by in house R script, using the density matrix data produced by deeptools (version 3.3.2).^[^ ^34^ ^]^

### Bioinformatic Analysis of Clinical Data

Overall survival analysis of gene expression and the correlation analysis in patient ccRCC tumors were carried out via the GEPIA platform (http://gepia.cancer‐pku.cn/). The log‐rank test was used for hypothesis evaluation. Values of p < 0.05 were considered as significantly different.

### Generation of Knockout or Knockdown Cell Lines

The single guide RNA (sgRNA) and short hairpins (shRNA) sequences were designed by using the GPP Web Portal (https://portals.broadinstitute.org/gpp/public/), provided by Broad Institute. The target sequences of sgRNAs and shRNAs are shown in Table S6 (Supporting Information). The sgRNAs were cloned in lentiCRISPRv2‐puro and shRNAs were cloned in PLKO.1‐puro (Addgene, #98 290). To construct knockout /knockdown cell lines, the lentiviral particles were generated by transfecting HEK293T cells with the indicated plasmid. Then the supernatant was used to infect the desired cells, and the cells were selected by puromycin.

### CRISPR‐dCas9‐KRAB Mediated Repression of Variant Enhancers

Site‐specific sgRNAs targeting enhancer loci were designed with publicly available filtering tools (https://zlab.bio/guide‐design‐resources) to minimize off‐target cleavage. The target sequences of sgRNAs were shown in Table S6 (Supporting Information). For CRISPR interference, sgRNAs were cloned into pLH‐spsgRNA2 (Addgene, #64 114) through the BbsI site according to the protocol recommended by Addgene. Lentivirus was generated by transfecting HEK293T cells with sgRNA expression cocktails or pHAGE dCas9‐KRAB‐MeCP2, together with helper plasmids, psPAX and pMD2G. Medium containing virus was collected at 48 or 72 h after transfection, and filtered with 0.45 µm filters (Millipore). Stable cell lines were generated by infecting with lentivirus expressing dCas9‐KRAB‐MeCP2 and sgRNAs, and then screened with puromycin and hygromycin for 48 h.

### Chromosome Conformation Capture (3C) Assay

Approximately 1 × 10^7^ cells were crosslinked with 1% formaldehyde for 10 min and quenched by glycine. The cells were washed with PBS and lysed in cell lysis buffer (10 mM Tris‐HCl, pH 7.5, 10 mM NaCl, 5 mM MgCl_2_, 0.1 mM EDTA, 1× complete protease inhibitor) at 4 °C for 30 min. Nuclei were collected after centrifugation at 400 × g at 4 °C for 5 min and removing the supernatant. The collected nuclei were digested with 400 U of DpnII restriction enzyme (NEB) at 37 °C overnight. The digested nuclei were then added with 100 U of T4 DNA ligase (NEB) and incubated for 4 h at 16 °C followed by 30 min at room temperature. The samples were then added with 300 µg of proteinase K, incubated at 65 °C overnight for de‐crosslinking, and purified with a DNA purification kit (TIANGEN DP214‐03). The relative crosslinking frequencies between the FABP5 enhancer and promoter were determined by qRT‐PCR. One primer in the enhancer (E1) and six primers in the promoter and gene body region (P1–6) were designed. The relative cross‐linking frequencies were calculated by normalizing to a primer pair (3C‐Ctrl) without crossing the DpnII cut sites. Assays were repeated at least three times. Data were shown as average values ± SD and p‐values were calculated using the student's *t*‐test. The sequences of primers are in Table S5 (Supporting Information).

### MTT Assay for Cell Proliferation

Cells were seeded on a 96‐well plate and cultured for 24, 48, 72, or 96 h. 5 µL MTT (5 µg/µL) was added into each well, and incubated for 4 h at 37 °C. Four hundred microliters of lysate buffer (50% DMF + 30% SDS, pH 4.7) was added into each well, followed by 4 h incubation at 37 °C. The absorbance at 570 nm was measured by a Microplate System. Assays were repeated at least three times. Data were shown as mean ± SD, and p value was calculated by the student's *t*‐test.

### Transwell Migration Assay

1.5×10^4^ 786‐O, 2×10^4^ 769‐P or 2×10^4^ A498 cells were loaded per transwell insert (24‐well insert; pore size, 8 µm; BD Biosciences) without serum or growth factors, and medium supplemented with 10% fetal bovine serum was used as a chemoattractant in the lower chamber. After 36 h of incubation, cells at the underside of the membrane were fixed in methanol for 10 min and stained with crystal violet (0.1% in 20% EtOH for 20 min). After washing, the remaining cells in the insert were removed by a cotton swab. Migrated cells were visualized by light microscopy and counted with ImageJ. Assays were repeated at least three times. Data were shown as average values ± SD of at least three representative experiments, and p value was calculated using student's *t*‐test.

### Immunoprecipitation and Western Blotting

Cells were harvested and lysed in high salt lysis buffer (20 mM HEPES, pH 7.4, 350 mM NaCl, 1% NP‐40, 0.5% TrintonX‐100) in the presence of proteinase inhibitors. The supernatant was incubated with Magnetic Beads‐conjugated Mouse anti DDDDK‐Tag (ABclonal) at 4 °C for overnight. The beads were pulled down and washed three times with lysis buffer. Then SDS loading buffer was added to the beads to release proteins for SDS‐PAGE and Western blotting. Cell lysates were prepared with SDS lysis buffer (50 mM Tris‐HCl pH 6.8, 4% SDS). Lysates were separated by SDS‐PAGE and transferred to nitrocellulose filter membranes blocked with 5% milk and incubated with primary antibody overnight at 4 °C. Then the blots were washed three times in TBS‐T, incubated with secondary antibodies at room temperature for 1 h, and detected by Clarity Western ECL Substrate (BIO‐RAD).

### H&E Staining and IHC Analysis

Tissue sections were deparaffinized, rehydrated, and treated for heat‐mediated antigen retrieval. Next, the sections were incubated in 3% H_2_O_2_ for 15 min at room temperature to quench the activity of endogenous peroxidase. After incubation in 3% BSA for 30 min, the sections were treated with primary antibody for FABP5 (dilution 1:400) or H3K27ac (dilution 1:1000) at 4 °C overnight. Then the sections were washed three times with PBS and treated for 30 min with horseradish peroxidase labeled goat‐anti‐rabbit IgG. After washed four times with PBS, the slide was visualized by DBA developer (Servicebio, G1211), and nuclei were stained with haematoxylin before examination by Olympus BX51 microscope.

### Nile Red Staining

Cells were cultured on the cover slips and fixed with 4%PFA for 15 min, and then washed twice in PBS. Added 5 µM Nile Red (MCE, HY‐D0718) in 200 µL PBS to the cover slips and incubated in the 37 °C Shaker for 30 min. Washed three times in PBS, then the slips were mounted with prolong anti‐fade kit (Invitrogen) and observed with confocal fluorescent microscopy.

### Acetyl‐Coenzyme A (CoA) Measurement

1×10^7^ cells were collected by centrifugation at 400 g, followed by ultrasonication and centrifugation at 18 000 g for 20 min at 4 °C. Supernatants (500 µL) were lyophilized and reconstituted in 100 µL ddH2O, vortexed at 650 rpm for 20 min at 4 °C, and centrifuged at 2000 g for 10 min at 4 °C. The supernatants were analyzed for acetyl‐CoA using UPLC‐MS/MS (ACQUITY UPLC‐Xevo TQ‐S, Waters) with MassLynx software (v4.1) for data processing and quantification against standard samples.

### Triglyceride (TG) Measurement

Triglyceride was measured using the TG Assay kit (NJJCBio, A110‐1‐1). In brief, 1×10^7^ cells were collected and centrifuged at 1000 rpm for 5 min, and removed the supernatant. the cell pellets were washed with PBS and resuspended with PBS. After ultrasonication, cell lysate (2.5 µL) was mixed with working solution (250 µL) in a 96‐well plate and incubated at 37 °C for 10 min. Finally, the absorbance was measured at 500 nm.

### Organoid Culture and Lentivirus Infection

CcRCC tumor and adjacent tissues were preserved in protection solution (advanced DMEM/F12 with 1% penicillin/streptomycin, 100 µg ml^−1^ Primocin, and 10 µM ROCK inhibitor Y‐27 632 dihydrochloride). After being washed with cold‐PBS buffer, tissues were cut into 1 mm pieces and transferred to 50 ml tube. Tissues were centrifuged at 500 g for 5 min, resuspended in 5 ml digestion solution (advanced DMEM/F12 with 1% penicillin/streptomycin, 100 µg ml^−1^ Primocin 10 µM Y‐27 632 dihydrochloride 0.1 mg ml^−1^ DnaseI, and 2 mg ml^−1^ collagenases type II), and incubated in 37 °C Shaker for 20 min. Tissues fragments were filtered through 70 µm cell strainer and centrifuged at 500 g for 5 min. The pellets were washed with cold‐PBS, and then resuspended with cold‐Matrigel. 20000 cells in 40 µl Matrigel were planted into 24‐well plates. Cell culture plates were put upside down in the 37 °C incubator for 40 min, and 500 µl ccRCC organoid culture medium (Table S7, Supporting Information) was added to each well and changed every 3–4 days.

Organoids passage and lentivirus infection were performed as the published protocol with minor modifications.^[^ ^21^ ^]^ Briefly, organoids were collected in cold‐PBS and resuspended with TrypLE Express for 5–10 min at 37 °C. 10 ml advanced DMEM/F12 was added to dilute the TrypLE Express. The organoids were centrifuged and resuspended with purified lentivirus and 8 µg ml^−1^ polybrene at 37 °C for 90 min. Then the organoids were centrifuged and resuspended with cold‐Matrigel. 500 organoids in 40 µl Matrigel were planted into each well of 24‐well plates and cultured for the desired time.

### Mice Subcutaneous Tumor Model

4×10^6^ 786‐O in 100 µL PBS with Matrigel were subcutaneously injected into the left flank of 5‐week‐old male BALB/C nude mice. The tumor size was monitored every 3 days at 40 days after injection. Mice were sacrificed at 80 days after injection.

### Mice Orthotopic Tumor Model

5‐week‐old male BALB/C nude mice were anesthetized and placed in a lateral position. 8–10 mm incisions were cut in the skin and muscle along the left flank of mice, and the kidneys were exposed. 7×10^5^ luciferase‐overexpressed 786‐O in 20 µL PBS were orthotopically injected into the left kidney with 30 G needles. The wound was sutured after surgery. The tumor size was monitored 3 months after injection by IVIS spectrum (PerkinElmer, USA) 10 min after intraperitoneal injection of 100 mL of D‐Luciferin potassium salt (15 mg mL^−1^). Mice were sacrificed at 5 months after injection.

### Animal Housing

The BALB/C nude mice were purchased from GemPharmatech Co., Ltd. All the mice were born and maintained under specific pathogen‐free (SPF) condition at 20–24 °C with a humidity of 40–70% and a 12/12‐h dark/light cycle (lights on at 7:00 AM, lights off at 7:00 PM), with free access of water and food (Animal Center of College of Life Sciences, Wuhan University).

### Ethics Approval and Consent to Participate

The patients’ tissues for ChIP‐seq and RNA‐seq were collected after surgery by the Department of Biological Repositories, Zhongnan Hospital of Wuhan University, Wuhan, China. RCC patients’ samples were collected from July 2019 to March 2021 regardless of age, gender, and tumor stage. The relevant clinical information is provided in Table S8 (Supporting Information). The patient study was performed in accordance with the Declaration of Helsinki and was approved by the Institutional Ethics Committee of Zhongnan Hospital of Wuhan University (approval number: 2 020 006). Written informed consents were obtained from individuals.

All the animal operations followed the laboratory animal guidelines of Wuhan University and were approved by the Animal Experimentation Ethics Committee of Wuhan University (Protocol NO. 14110B).

### Statistics and Reproducibility

For experiments other than NGS sequencing, at least three biological replicates for each experiment were performed. Data were presented as mean values ± SD or SEM shown in figure legends. Statistical analysis was performed using a two‐sided Student *t*‐test. The p‐value was either labeled on the corresponding items or listed in the legends.

### Availability of Data and Materials

All the reagents presented in this paper were available upon request. ChIP‐Seq and RNA‐seq data for RCC patients were accessible at GSA‐Human of CNCB (China National Center for Bioinformation, https://www.cncb.ac.cn/; Acc. NO. HRA010247).

## Conflict of Interest

The authors declare no conflict of interest.

## Author Contributions

Z.Y. and Q.X.H. contributed equally to the work. Y.Z. and H.Q.X. performed most of the experiments. W.M.L. and C.J.D. did data analysis. S.J., Y.N.R., J.L., Q.W.L., C.J., W.Z., and X.X.Y. helped in experiments. C.Z.Y. and Y.W.M. collected tissues for organoids. X.Y. and Q.K.Y. collected tissues for sequencing. X.Y., L.L.Y., C.M.K., and J.L.G. discussed the project and provided suggestions. C.M.K., J.L.G., and W.M. directed the study. H.Q.X., Y.Z., W.M.L., and W.M. wrote the manuscript. X.Y., L.L.Y., C.M.K., and J.L.G. edited the manuscript.

## Supporting information



Supporting Information

Supporting Information

## Data Availability

The data that support the findings of this study are available from GSA‐Human of CNCB. Restrictions apply to the availability of these data, which were used under license for this study. Data are available at https://www.cncb.ac.cn/ with the permission of GSA‐Human of CNCB.
