# Supplementary material for: A Feedback Loop Between Fatty Acid Metabolism and Epigenetics in Clear Cell Renal Carcinoma
Source: Adv Sci (Weinh). 2025 May 20;12(28):e04532. doi: 10.1002/advs.202504532 (PMC12302539; doi:10.1002/advs.202504532)
Supplement: Supplementary file 1 — Supporting Information [file ADVS-12-e04532-s001.pdf]

ADVANCED SCIENCE
Open Access

Supporting Information

for *Adv. Sci.*, DOI 10.1002/advs.202504532

A Feedback Loop Between Fatty Acid Metabolism and Epigenetics in Clear Cell Renal Carcinoma

Zhou Ye, Qi-Xin Hu, Ming-Liang Wei, Ji-Dong Chen, Jia Shi, Ning-Rong Yang, Lu Jiang, Jian Chen, Zhi-Yuan Chen, Wei-Min Yu, Yu Xiao, Kai-Yu Qian, Zilin Xu, Zhong Wang, Wen-Lu Qi, Xin-Yi Xiao, Yu-Yu Duan, Yong Xiao, Lian-Yun Li, Lin-Gao Ju*, Ming-Kai Chen* and Min Wu**

Sup. Figure S1

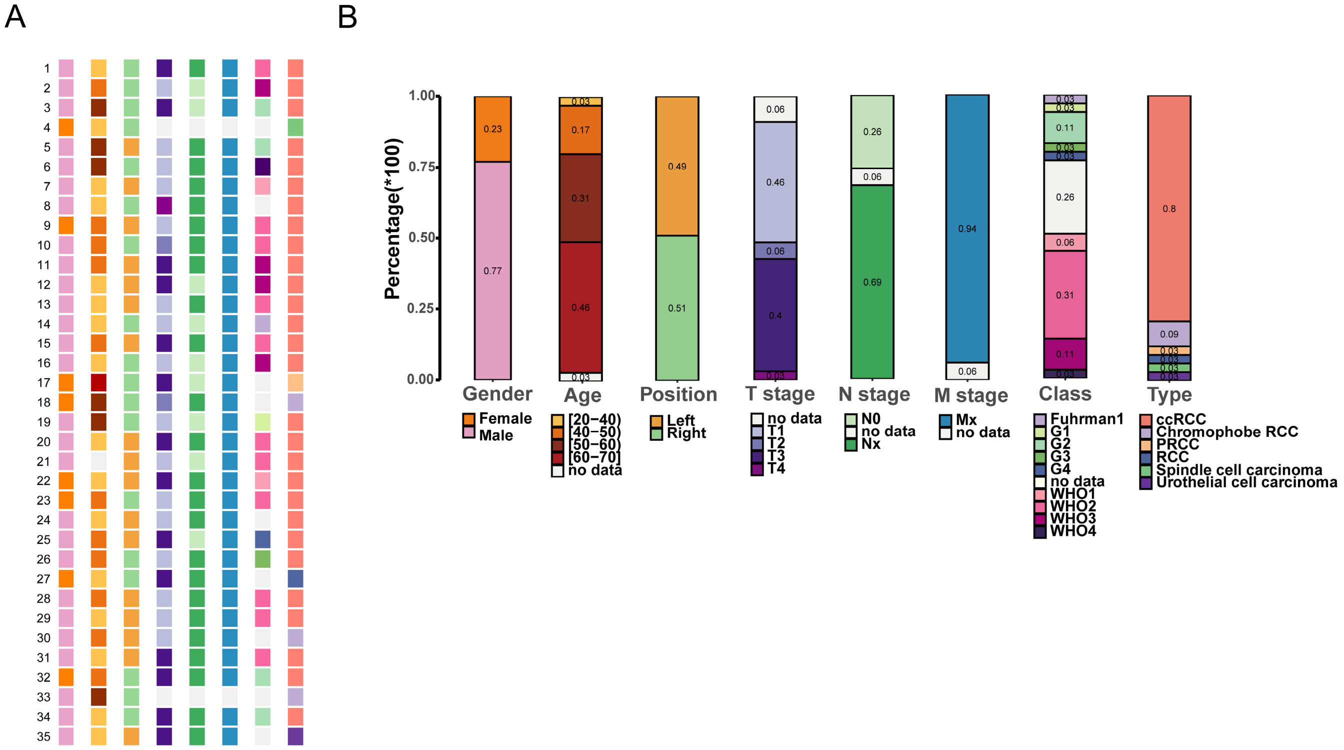

Sup. Fig. S1 Information of collected RCC tissues. A. The clinical information of all 35 RCC patients in our study. **B.** Bar plot showing the clinical information statistics of RCC patients.

Sup. Figure S2

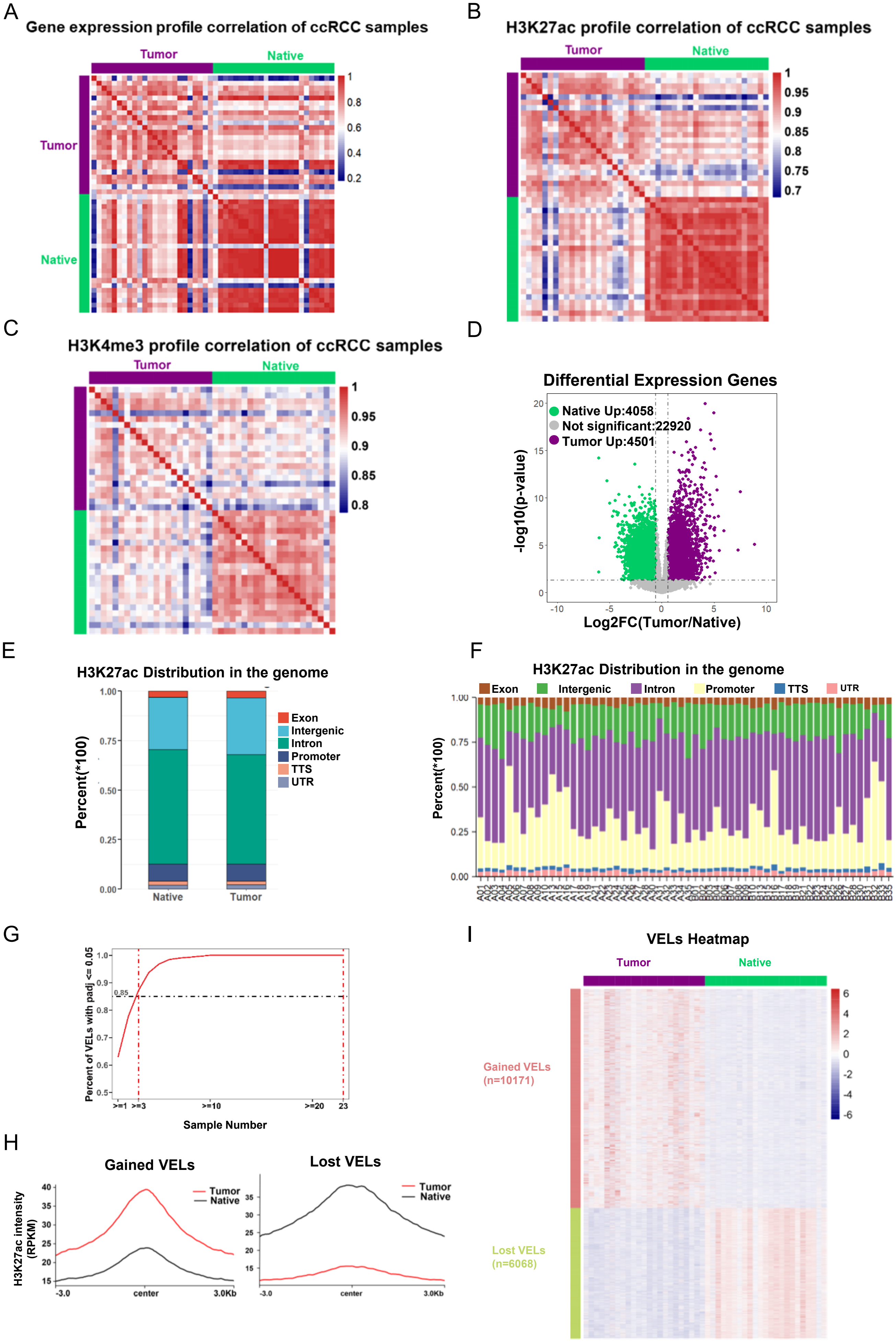

Sup. Figure S2

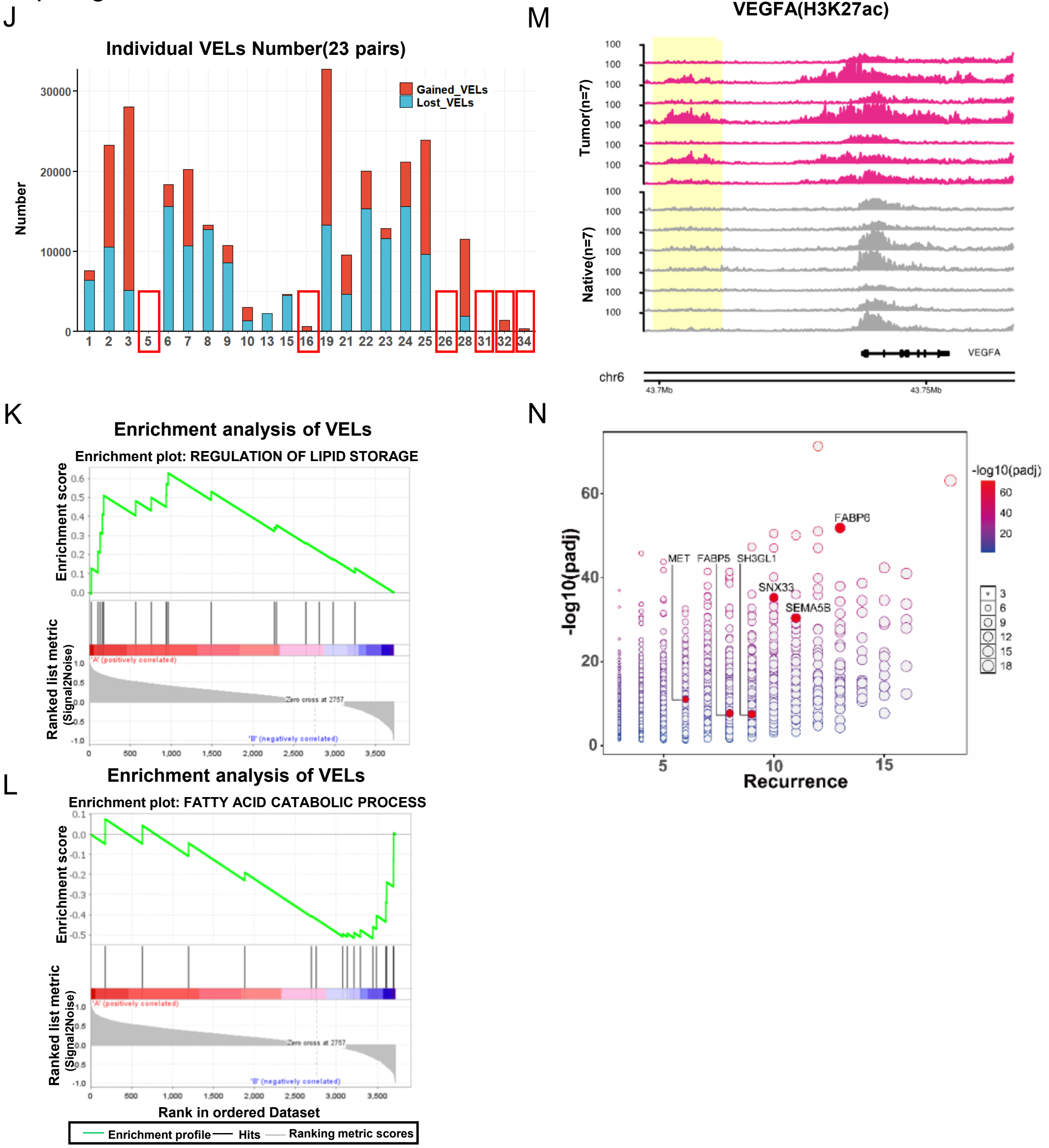

Sup. Fig. S2 Profiling of variant enhancers of ccRCC patient tissues. A-C. Heatmap for the spearman correlation of gene expression profiles (A), H3K27ac (B) and H3K4me3 (C) among tumor and native tissue samples from all CRC patients. **D.** DEG analysis comparing tumor and native tissues. Purple dots represent high expressed genes in tumors, green for high expressed genes in native tissues, and grey for genes not significantly changed. **E.** Genomic distribution of H3K27ac signals in tumor and native tissues from ccRCC patients. **F** Genomic distribution of H3K27ac signals in tumor and native tissues of individual patients. **G.** The required recurrence for VELs meeting statistical significance (p.adj < 0.05). The vertical red line at left highlights the recurrence of VELs at 3 when achieve the cut-off (0.85, black dashed line) of significant percentage, and the red lines at right highlights the highest recurrence of VELs. p.adj indicates the BH adjusted t-test p-value. **H.** The average H3K27ac signal (RPKM) at the regions of gain VELs (left) and lost VELs (right) in tumor and native tissues. **I.** Heatmap of relative H3K27ac signals of gain and lost VELs in tumor and native tissues. **J.** Numbers of gained or lost VELs from each ccRCC patient. **K&L.** Enrichment analysis of genes associated with the VELs in the tumor versus native tissues. **M.** Representative H3K27ac tracks of the gain VEL on *VEGFA* loci. **N.** The genes associated with VELs ranked by recurrence and p-value. Red dots represent the enhancers functional verified after screening.

Sup. Figure S3

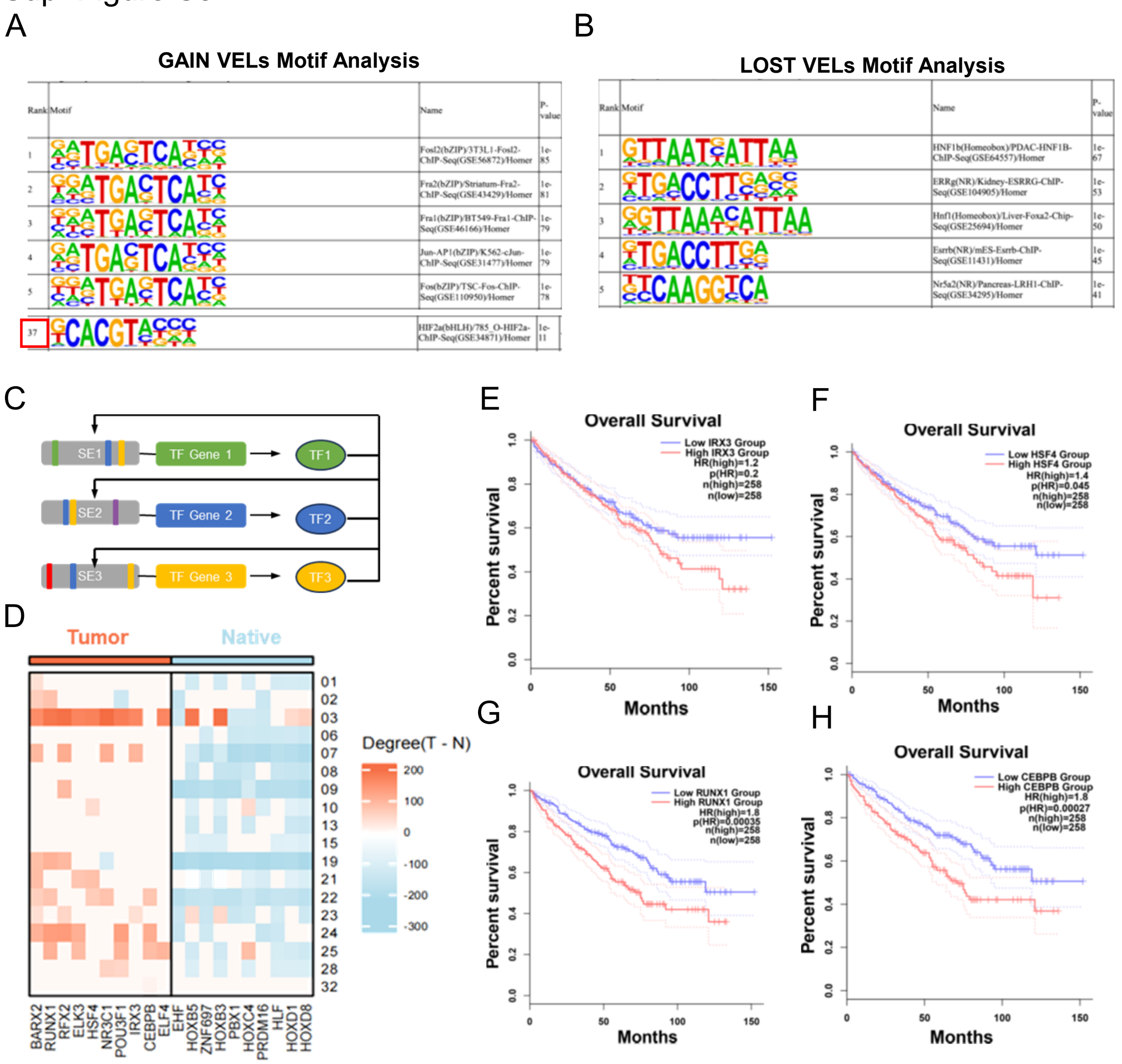

Sup. Fig. S3 Prediction of functional transcription factors in ccRCC. A&B. DNA motifs enriched within nucleosome-free regions (NFRs) of tumor gain (A) or lost (B) VELs determined by HOMER motif analysis. **C.** Diagram of core regulatory circuitry (CRC) prediction model. **D.** Heatmap of transcription factors by predicted CRC model in tumor and native tissues. Top 10 tumor and native-specific TFs were listed. **E-H.** Overall survival rates of KIRC patients with high or low *IRX3* (E), *HSF4* (F), *RUNX1* (G) or *CEBPB* (H) expression based on TCGA datasets, log-rank test, n= 516 patients.

Sup. Figure S4

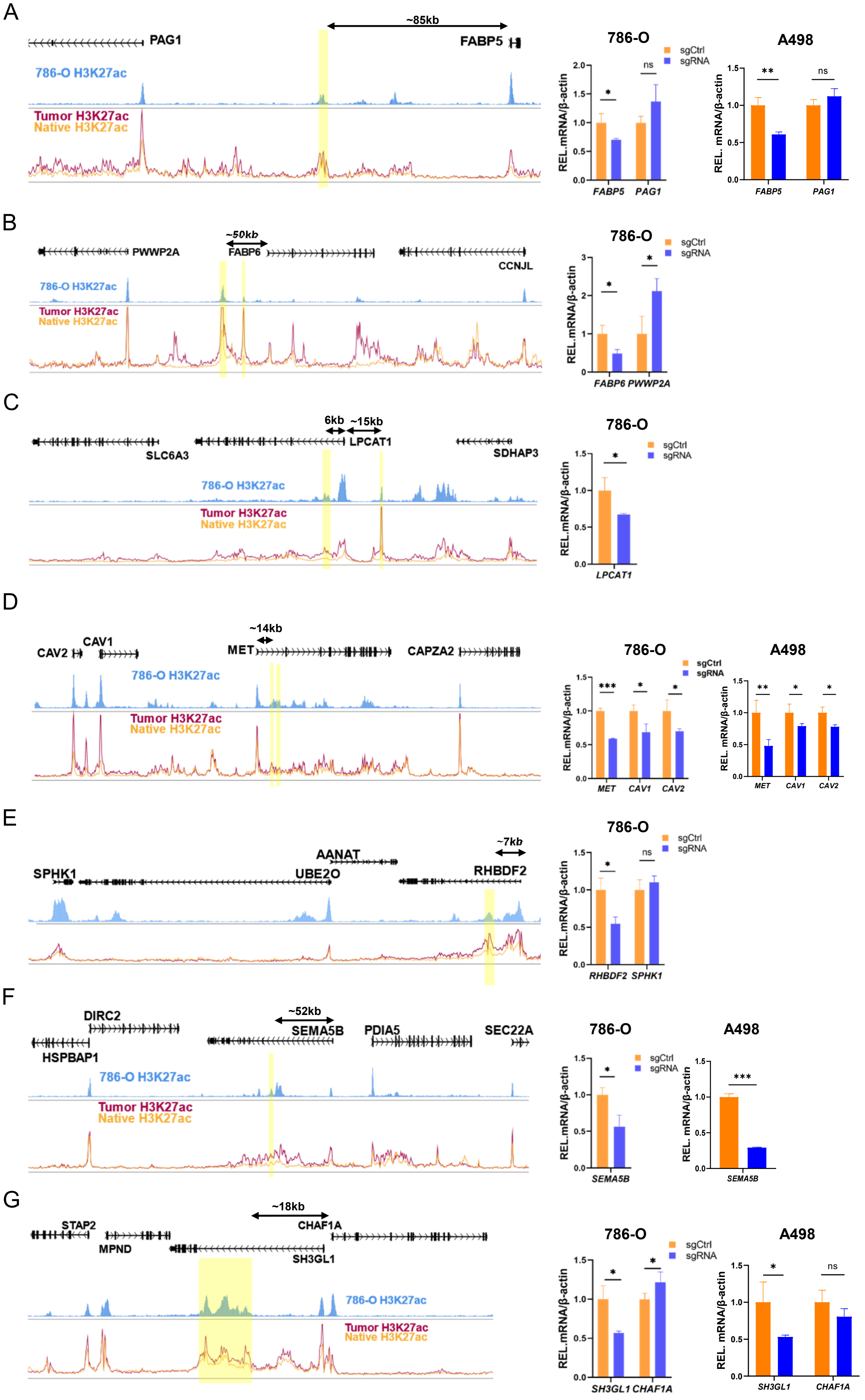

Sup. Figure S4

H

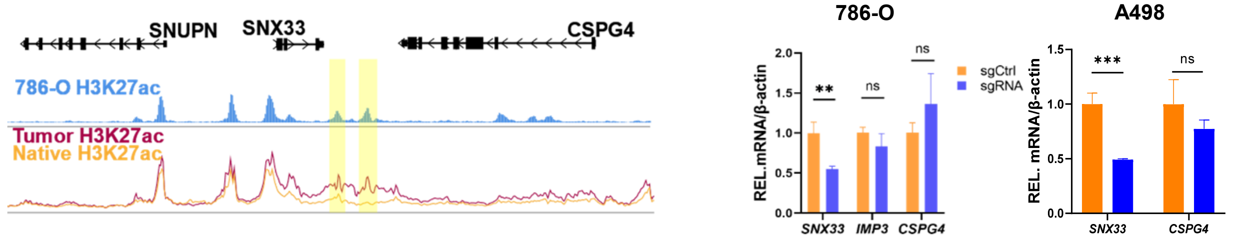

I

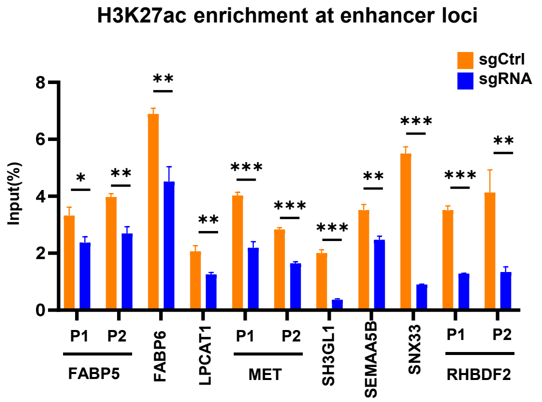

J

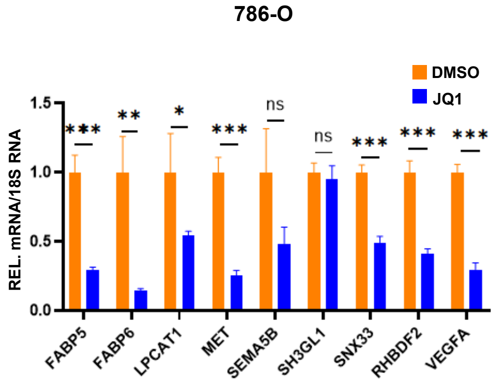

Sup. Fig. S4 Validation of functional tumor-specific enhancers. A-H The design of sgRNA targeting the gain VELs were shown on the left, and their effects on gene transcription detected by quantitative PCR were shown on the right. The highlight region represent the target genome site for sgRNAs. Multiple cell lines were used for the VELs functioning in other assays. n = 3 for all samples. Statistical analysis was performed using a two-sided student t test. **I.** H3K27ac enrichment on *FABP5*, *FABP6*, *LPCAT1*, *MET*, *SH3GL1*, *SEMA5B*, *SNX33* and *RHBDF2* enhancer loci in control and sgRNA group. **J** Relative mRNA level of indicated genes in 786-O treated with 5 μM JQ1 for 36h. Bars represent mean values ± SD, * $p < 0.05$, ** $p < 0.01$, *** $p < 0.001$, two-sided t-test.

Sup. Figure S5

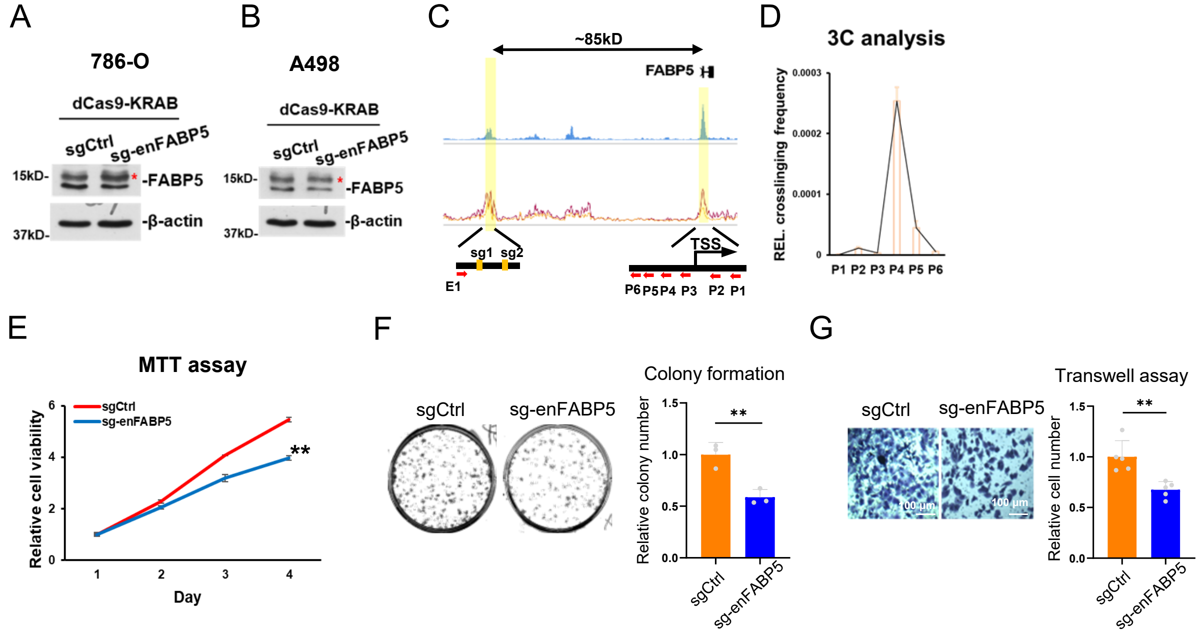

Sup. Fig. S5 *FABP5* enhancer is critical for ccRCC. A&B. FABP5 expression in dCas9-KRAB 786-O (A) or A498 (B) cells transfected with control sgRNA or *FABP5* enhancer sgRNA. **C.** The genome browser view showing H3K27ac enrichment in the region of *FABP5* promoter and enhancer. The red arrows indicate the loci of 3C primers. The yellow bars indicate the loci targeted by dCas9-KRAB sgRNAs. **D.** Relative crosslinking frequency of *FABP5* promoter and enhancer performed by quantitative PCR (n=3). **E-G.** MTT assay (E), colony formation assay (F) and transwell assay (G) of A498 cells with repressed *FABP5* enhancer. Bars represent mean values ± SD, * $p < 0.05$, ** $p < 0.01$, *** $p < 0.001$, two-sided t-test.

Sup. Figure S6

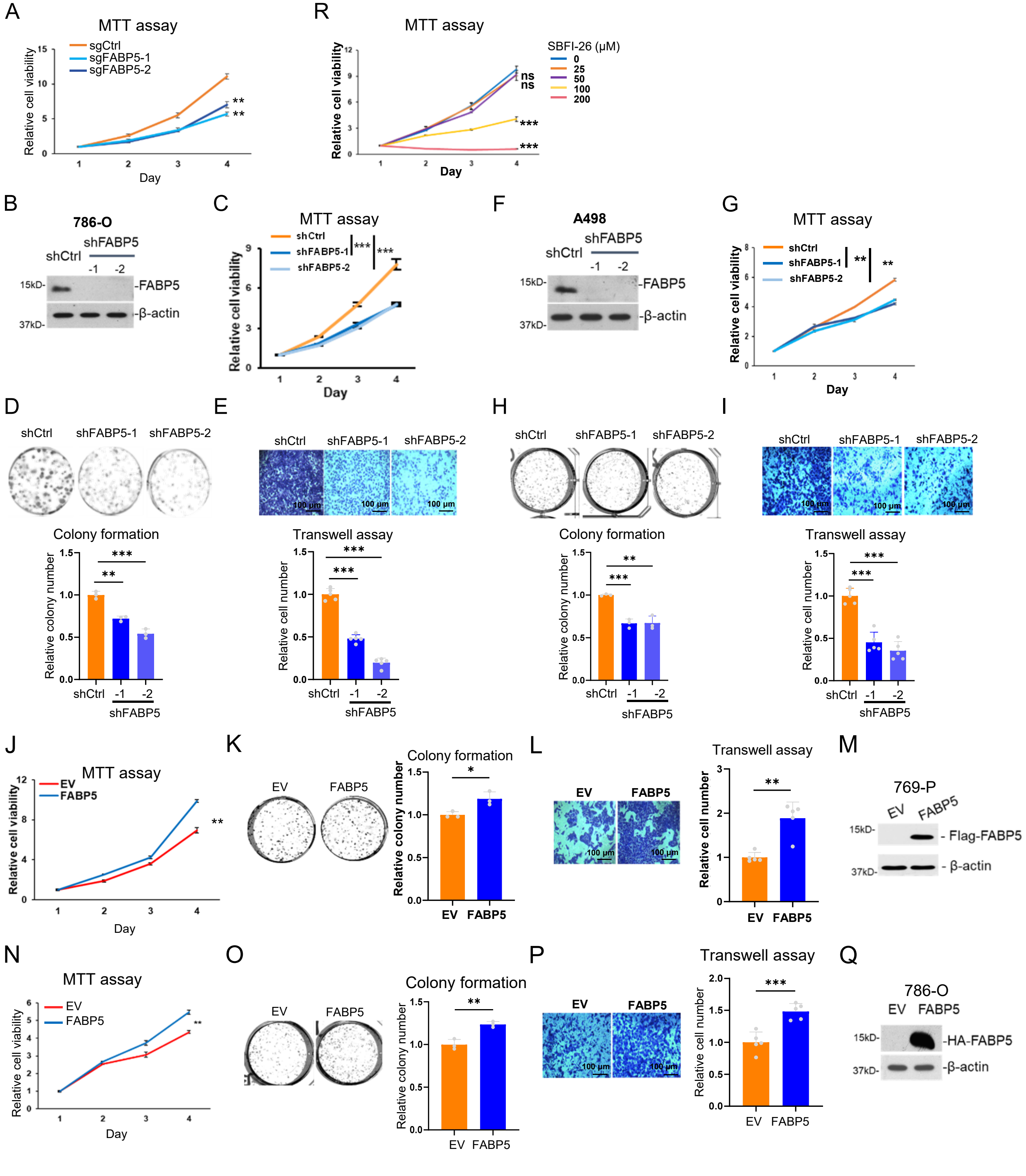

Sup. Fig. S6 The critical roles of FABP5 for CCRCC. A. MTT assay of control and *FABP5*-KO 769-P cells. **B-E.** Protein level detected by western blotting (B), cell proferilation detected by MTT assay (C), colony formation (D) and transwell assay (E) of control and *FABP5* knockdown in 786-O cells. **F-I.** Protein level (F), MTT assay (G), colony formation (H) and transwell assay (I) of control and *FABP5* knockdown in A498 cells. **J-M.** MTT assay (J), colony formation (K), transwell assay (L) and protein level (M) of control and *FABP5* stably expressed 769-P cells. **N-Q.** MTT assay (N), colony formation (O), transwell assay (P) and protein level (Q) of control and *FABP5* stably expressed 786-O cells. **R.** MTT assay of 786-O cells treated with a dose concentration of SBFI-26(25, 50, 100, 200 μM) for 4 days. n=3. Bars represent mean values ± SD, * $p < 0.05$, ** $p < 0.01$, *** $p < 0.001$, two-sided t-test.

Sup. Figure S7

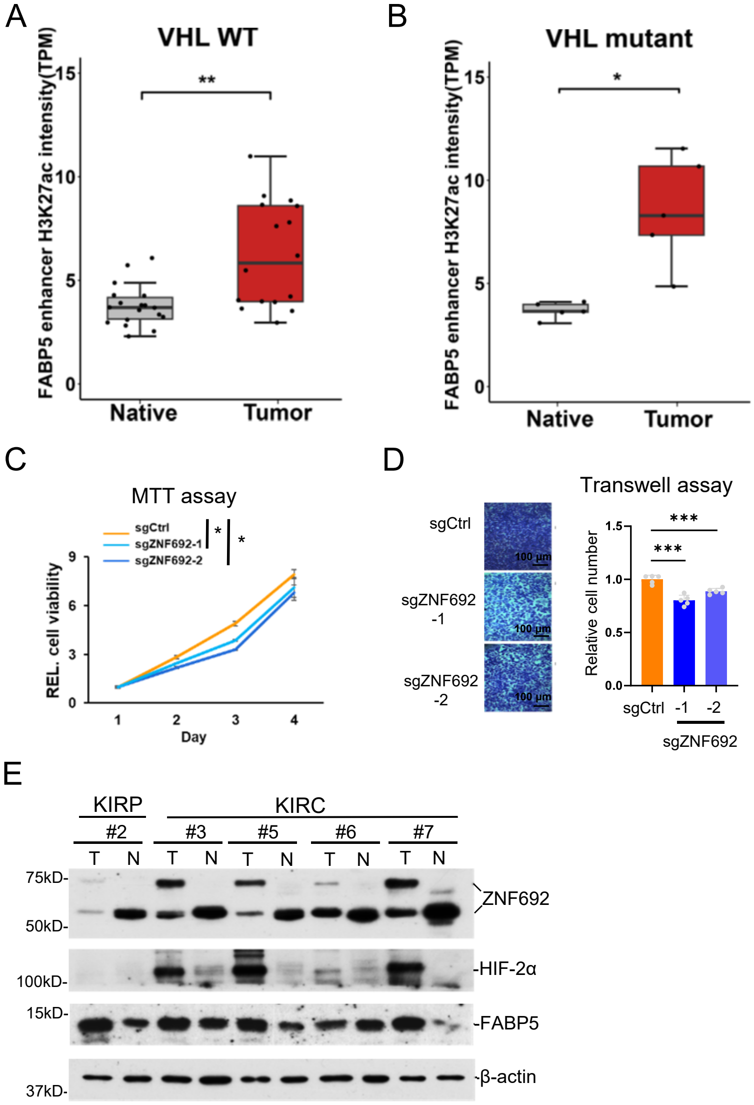

Sup. Fig. S7 Regulation of FABP5 by HIF family proteins and ZNF692. A&B. H3K27ac enrichment on *FABP5* enhancers in the normal and ccRCC tissues with wild-type VHL (A) or mutant VHL (B). n=18 for WT, 6 for mutant. **C-D.** MTT assay (C) and transwell assay (D) of control and *ZNF692*-KO 786-O cells. **E.** ZNF692 and HIF-2α expression in the kidney cancer patient tissues of Fig. 3I. Bars represent mean values ± SD, * $p < 0.05$, ** $p < 0.01$, *** $p < 0.001$, two-sided t-test.

Sup. Figure S8

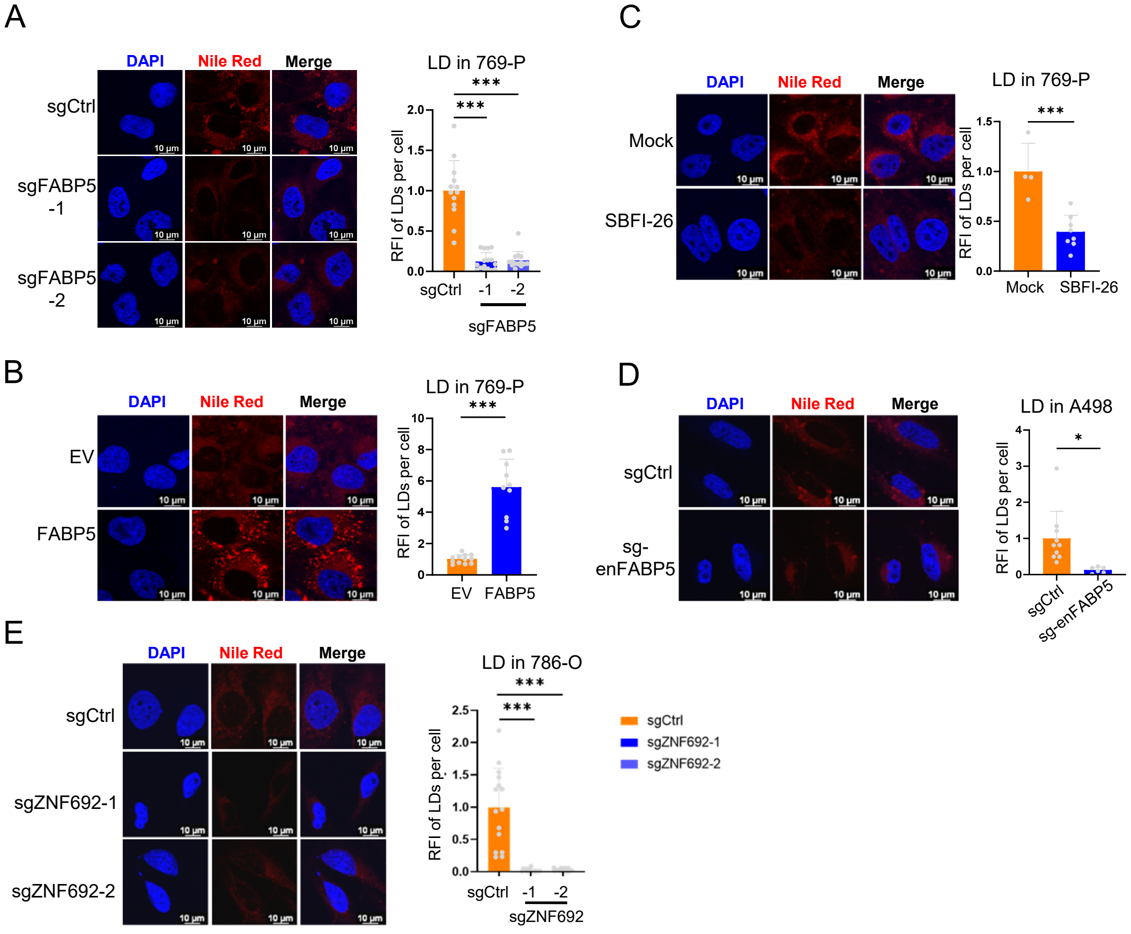

Sup. Fig. S8 FABP5 is critical for LD formation in ccRCC cells. A. Representative fluorescence images and quantitative data of control and *FABP5*-KO 769-P cells stained for LDs (red) and nuclei (blue). **B.** Representative fluorescence images and quantitative data of control and *FAPB5*-OE 769-P cells stained for LDs (red) and nuclei (blue). **C.** Representative fluorescence images and statistical data of 769-P cells treated with 100 μM SBFI-26 for 36h, stained for LDs (red) and nuclei (blue). n= 4 (DMSO), 8 (SBFI-26). **D.** FABP5 enhancer was repressed in A498 cells. Representative fluorescence images and statistical data were shown. n= 10 (sgCtrl), 6 (sg-enFABP5). **E.** ZNF692 stable knockdown cells shown in Fig. 4I was stained for lipid droplets (red) and nuclei (blue). Bars represent mean values ± SD, * $p < 0.05$, ** $p < 0.01$, *** $p < 0.001$, two-sided t-test.

Sup. Figure S9

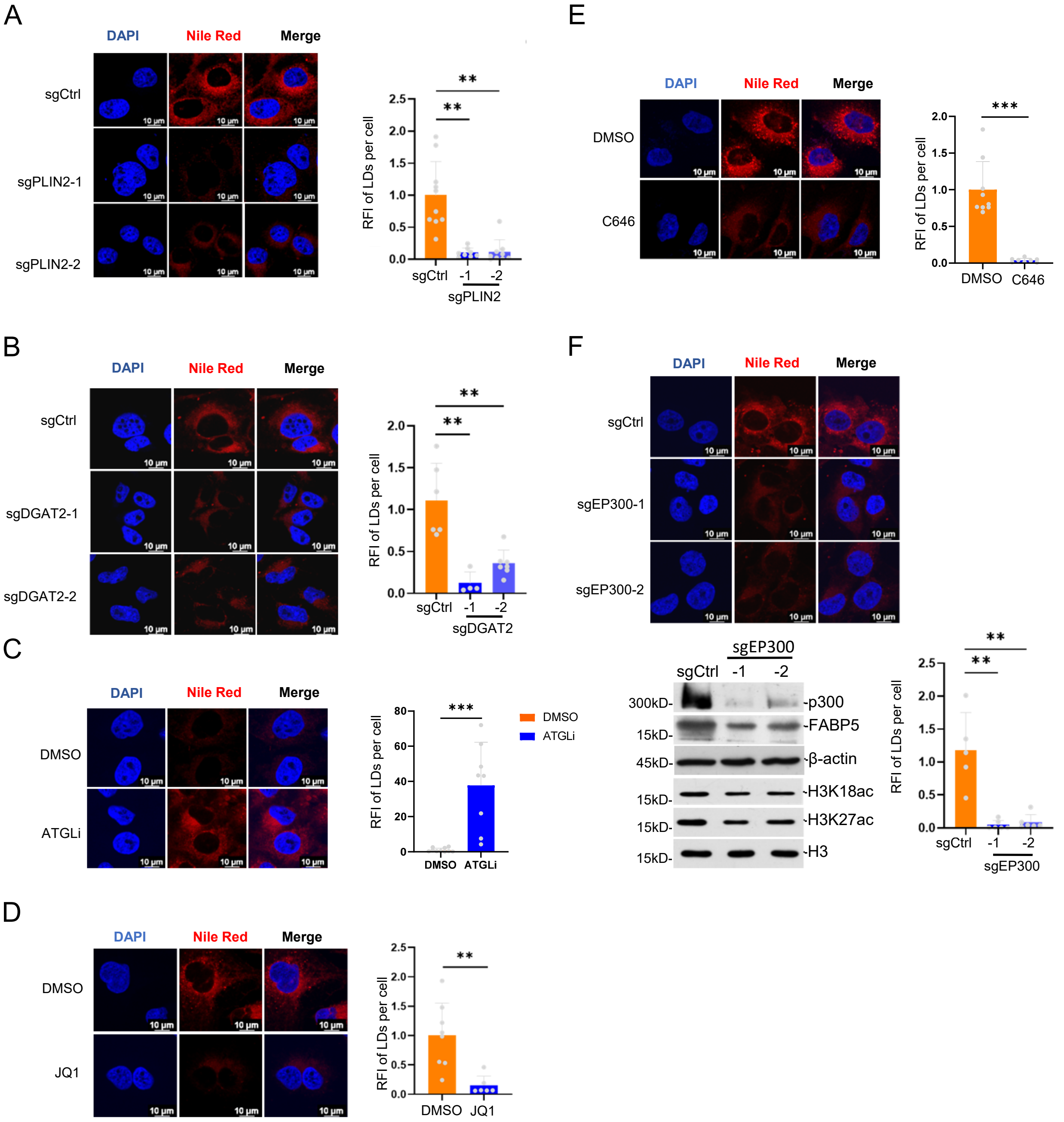

Sup. Fig. S9 Crosstalk between lipid metabolism and epigenetics in ccRCC. A&B. PLIN2 (I) or DGAT2 (J) was knocked down in 769-P cells and fluorescent staining was performed, Nile red for LDs, DAPI for DNA. **C.** Representative fluorescence images and statistical data of 769-P cells treated with 10μM ATGL inhibitor (ATGLi) for 36h, then stained for LDs (red) and DNA (blue). **D.** Representative fluorescence images and statistical results of 786-O cells treated with 5μM JQ1 for 36h, followed by staining for LDs (red) and DNA (blue). n= 8 (DMSO), or 6 (JQ1). **E.** Representative fluorescence images and statistical analysis of 786-O cells treated with 5μM C646 for 36h, followed by staining for LDs (Red) and DNA (blue). n= 9 (DMSO), or 8 (C646). **F.** p300 was knocked down in 769-P cells, and fluorescent staining, western blotting and statistical analysis were performed as indicated. Bars represent mean values ± SD, * $p < 0.05$, ** $p < 0.01$, *** $p < 0.001$, two-sided t-test.
